# Machine Learning Approach for Cardiovascular Disease Risk Stratification Using Ensemble Classifiers and Dimensionality Reduction

**DOI:** 10.3390/life16101724

**Published:** 2026-10-15

**Authors:** Alejandro Posada-Castellanos, Miguel Angel Gomez-Aristizabal, Carlos Manuel Orrego-Franco, Elisabeth Restrepo-Parra, Luis Eduardo Lopez-Betancur

**Affiliations:** 1 Departamento de Física y Química, Universidad Nacional de Colombia, Sede Manizales, Km. 9 vía al Aeropuerto, Manizales 170001, Colombia; alposadaca@unal.edu.co (A.P.-C.); corregof@unal.edu.co (C.M.O.-F.); erestrepopa@unal.edu.co (E.R.-P.); 2 Physics-Chemistry-Mathematics (PCM) Computational Applications, Universidad Nacional de Colombia, Sede Manizales, Km. 9 vía al Aeropuerto, Manizales 170001, Colombia; 3 INNTERFAZ CDT, Campus La Nubia, Km. 9 vía al Aeropuerto, Manizales 170001, Colombia; lelopezb@unal.edu.co

**Keywords:** cardiovascular disease risk stratification, machine learning, ensemble classifiers, dimensionality reduction, clinical decision support

## Abstract

Cardiovascular disease (CVD) remains a leading cause of morbidity and mortality worldwide, motivating data-driven tools that complement established clinical risk criteria. We present a two-pathway machine learning framework for CVD risk assessment. Pathway I is a brief rule-learnability verification, a four-level risk label is generated by a deterministic MEWS/Framingham-inspired scoring rule, and because the label is a deterministic function of the inputs, its near-perfect recovery by the classifiers is only a consistency check, not predictive evidence. Pathway II, the focus of the study and the main contribution of this work, is a hybrid pipeline that combines unsupervised preprocessing (K-Nearest Neighbors imputation, PCA/UMAP dimensionality reduction, K-Means phenotyping) with supervised ensemble classifiers (Random Forest, XGBoost, LightGBM), trained on an ≈70,000-record Kaggle cohort and externally validated on an independent 219-patient cohort using the same prevalent-CVD definition. On the external cohort the six pipelines performed comparably (accuracy 70–74%, external-cohort AUC 0.82–0.85); paired McNemar tests showed no significant difference between UMAP- and PCA-based pipelines (all p>0.5), and a power analysis indicates the cohort is too small to distinguish them, so no configuration is claimed to be superior. An unsupervised phenotype analysis is included as a descriptive, hypothesis-generating step; its association with the outcome is statistically significant but of negligible effect size. The framework offers a reproducible, clinically interpretable methodology for CVD risk categorization.

## 1. Introduction

The recent proliferation of Artificial Intelligence (AI) and Machine Learning models has led to the development of a multitude of analysis paths by categorizing risk factors related to cardiovascular disease and establishing a risk curve for patients with related comorbidities. There is also a wide range of treatment options and risk identifiers that have led to establish a set of apparently disconnected variables that play an important role in CVD diagnostic options. Machine learning (ML) models provide a data-driven approach to discriminating between competing treatment strategies. One set of general variables included for Cardiovascular clinical purposes consists of age, gender, height, and weight. Another set focuses on patient lifestyle such as smoking status, alcohol intake, and physical activity which contributes indirectly to adverse effects on specialized risk variables. The wide range of machine learning models are of great use in filtering these variables and establishing specific criteria for non-linear analysis, data categorization, and the complex correlations found in variable behavior and their consequences for final risk outcomes; for instance, they can be used to predict long-term outcomes in patients with *recurrent pericarditis*, as demonstrated in [1]. The designed and implemented software pathways are intended to represent a clinical decision support system based on a collection of free available datasets and data preprocessing techniques. The system generates risk group classification for patients with cardiovascular disease (CVD), suggesting lifestyle modifications and, when necessary, urgent alerts advising them to contact their primary health institution if a critical risk indicator is detected. These data are processed and analyzed by machine learning models to continually refine risk assessments and display predicted outcomes. Beyond its role in the present analysis, the trained pipeline is currently being explored as the predictive foundation for a home-based diagnostic application, a direction still in early development that would extend cardiovascular risk stratification beyond clinical settings into everyday patient use. The integrated use of Artificial Intelligence (AI) toolsets facilitates the development of new technologies focused on improving symptom recognition and risk evaluation pathways for cerebro-cardiovascular diseases. AI is transforming diagnostic paradigms and treatment protocols, enhancing precision and equitably improving the quality of clinical care. Its application extends beyond early detection; it also encompasses continuous patient monitoring and treatment personalization, thereby optimizing clinical outcomes and interpretations related to CVD [2]. Furthermore, AI’s capacity to process vast datasets enables the detection of critical patterns that trigger timely clinical interventions, ultimately reducing hospitalizations and preventing complications associated with a CVD diagnosis [3]. Successful applications of AI now span multiple cardiology subfields; in cardiac electrophysiology, for instance, comprehensive reviews document its expanding role in diagnosis, risk stratification, and treatment guidance [4].

**Machine learning inside CVD knowledge:** Machine Learning (ML) algorithms can differentiate which variable or set of variables is/are the most pragmatic in risk prediction [5]. The growing armamentarium of preventive therapies requires critical evaluation, particularly regarding their efficacy and their integration with machine learning techniques, as well as their costs, availability, and clinical utility. It has been proven that automated learning algorithms, such as Support Vector Machines (SVM), Neural Networks, and gradient boosting methods including XGBoost and LightGBM, outperform traditional risk evaluation models. These advanced methods provide more reliable predictions for critical clinical outcomes, such as risk assessments related to myocardial infarction and heart failure [6]. These models are currently powerful tools for classifying and analyzing the multiple variables associated with cardiovascular risk. To achieve the high levels of accuracy and reliability required by clinical personnel, data preprocessing is essential. Techniques such as feature scaling, dimensionality reduction, and normalization are critical for data optimization. This process must account for diverse input types, including numerical values, string variables, and categorical indicators [7]. The present model is built on previously collected clinical records drawn from openly available Kaggle datasets [8,9,10]; through a hybrid methodology that combines unsupervised preprocessing with supervised classification, the system learns from these historical data to issue risk alerts for both healthy individuals and patients who require ongoing monitoring.

**Supervised vs Unsupervised Learning:** Within the ecosystem of machine learning frameworks, many models rely on available data to generate predictions through training on labeled datasets. However, instances occur where a dataset lacks a specific output indicator or "ground truth," significantly complicating the machine’s ability to identify the target variable. In such cases, the researcher must either rely on the user’s post hoc interpretation of the patterns found or restrict the analysis to datasets containing clear, predefined output indicators. Machine Learning systems can be classified according to the amount and type of supervision they get during training [11]. We used two of the four main categories coupled together: supervised learning and unsupervised learning. Supervised learning refers to a class of models where the training process utilizes datasets containing the desired outputs. In the context of Cardiovascular Disease (CVD) analysis, this involves feature vectors, comprising patient clinical data with a specific target column indicating risk levels. These are typically encoded as binary values, where 0 represents low risk and 1 indicates high risk. This approach allows the algorithm to learn the specific physiological thresholds and non-linear interactions within the patient data that correlate with adverse cardiovascular events. The datasets utilized in this study were obtained from publicly available Kaggle repositories [8,9,10]. The first dataset was designated as the training cohort after imposing biological boundary limits leaving the set of 55,779 patients after, whereas the third dataset was used as a small validation cohort. Within this supervised framework, Random Forest, LightGBM and XGBoost were selected to train the diagnostic criteria for classifying patients into low and high -risk levels for a given CVD. Specifically, the Light Gradient Boosting Machine (LightGBM) framework was integrated due to its exceptional computational efficiency when processing heterogeneous datasets that merge dense numerical variables with discrete categorical indicators. This native capacity to manage mixed-type feature spaces without requiring memory-intensive array expansions is a particularly relevant asset for the clinical cohorts examined in this study [12]. Conversely, unsupervised learning techniques process unlabeled datasets where a target output is unavailable, essentially allowing the system to learn patterns without a teacher [11]. Notable algorithms in this category include clustering methods such as K-Means, DBSCAN, and Hierarchical Cluster Analysis (HCA) as well as dimensionality reduction and visualization tools, including Principal Component Analysis (PCA), t-SNE, Locally Linear Embedding (LLE), and Uniform Manifold Approximation and Projection (UMAP).

There exists a significant limitation in current clinical decision support systems, which is the inherent trade-off between model interpretability and predictive performance. This issue, which remains critical in medical contexts, is often overlooked in the implementation of contemporary machine learning models [13]. To avoid presenting high-complexity numerical results without clarifying, the methodology contextualizes data by generating clear probability labels of high risk group alerts. This ensures that risk stratification is communicated effectively to both patients and healthcare professionals, thereby supporting informed clinical decision-making. The pathways moves beyond presenting raw numerical probabilities by providing meaningful clinical context. Specifically, they communicate risk levels in a clear, accessible format for both patients and the healthcare professionals responsible for final decision-making. Additionally, a comprehensive review of the mathematical frameworks underpinning this study, encompassing both the supervised learning models and dimensionality reduction techniques, is provided to elucidate the formal functions, definitions, and underlying mechanisms governing the algorithms. This detailed theoretical foundation can be found in the Appendix A, Appendix B, Appendix C, Appendix D, Appendix E and Appendix F.

The supplementary theoretical material is organized as follows: KNN preprocessing in Appendix A, Random Forest in Appendix B, XGBoost in Appendix C, LightGBM in Appendix D, PCA in Appendix E, and UMAP in Appendix F.

The main objective of this study is to develop and evaluate a two-pathway machine learning framework for cardiovascular risk stratification that combines a clinically grounded, rule-based labeling scheme (Pathway I) with a hybrid unsupervised-preprocessing plus supervised-classification pipeline (Pathway II), thereby addressing the interpretability performance trade-off discussed above. Whether dimensionality reduction techniques preserve or dilute clinically meaningful risk signal remains an open question in the literature, with linear (PCA) and manifold-based (UMAP) approaches offering competing trade-offs between interpretability and structural fidelity that have not been consistently resolved for tabular clinical data. The scientific questions are therefore distinct for each pathway: Pathway I asks whether an explicit clinical scoring rule can be recovered and deployed by standard classifiers, whereas Pathway II asks whether dimensionality reduction preserves the discriminative signal needed to classify prevalent CVD on an independent cohort. Our principal findings are that (i) the deterministic rule is recovered almost exactly, and (ii) on the external 219-patient cohort [10] the six ensemble reduction pipelines perform comparably, with paired McNemar tests showing no statistically significant difference between UMAP- and PCA-based variants. Accordingly, we do not claim superiority of either reduction technique; we report the framework as a reproducible, interpretable decision-support methodology and identify longitudinal outcome data and prospective validation as the next steps toward genuine prognostic use.

## 2. Materials and Methods

The proposed methodology implements a dual-pathway clinical risk assessment architecture. This framework systematically bridges traditional biostatistical guidelines with advanced data-driven models: the first pathway constructs an explicit four-level clinical risk label through a deterministic scoring rule inspired by the Modified Early Warning Score (MEWS) and Framingham-type cardiovascular risk factors, detailed later in this section while the second pathway relies on a binary target variable to train the binary hybrid machine learning pipeline. To train and validate the predictive models, the pipeline utilizes an open-access clinical cohort sourced from Kaggle [8], which comprises the largest number of records and serves as the primary training dataset. The dataset comprises ten baseline physiological, clinical, and lifestyle input features (age, height, weight, biological sex, systolic blood pressure (ap_hi), cholesterol stratification, glucose level, smoking status, alcohol consumption, and physical activity), together with one binary outcome label, the cardiovascular-disease group (*cardio*). The ten features are the model inputs; the cardiovascular-disease group is the target label, not a feature. Diastolic blood pressure (ap_lo) was recorded in the primary cohort but was excluded from all downstream modeling and validation (it is unavailable in the external cohort); it is therefore not one of the ten modeling features. Crucially, the dataset includes an explicit binary target variable indicating the documented presence or absence of a previously diagnosed cardiovascular pathology, serving as the foundational ground truth for pipeline calibration and evaluation.

**First pathway:** The first pathway establishes a clinically grounded multi-level risk stratification framework operating in parallel to the binary hybrid pipeline. Given that the training Kaggle cohort [8] provides only a binary outcome variable indicating the documented presence or absence of previously diagnosed cardiovascular pathology, an explicit four-level risk label

(1) y∈{0,1,2,3},

corresponding to low, moderate, high, and critical risk, respectively, is constructed through a deterministic scoring rule inspired by the Modified Early Warning Score (MEWS) and Framingham-type cardiovascular risk factors.

The composite clinical risk score s(x) is defined as

(2) $$\begin{array}{cc} s(x)=\; & s_{\mathrm{SBP}}\left(x\right)+s_{\mathrm{glu}}\left(x\right)\\ \; & +2\cdot 1[\mathrm{stroke}]+1[\mathrm{heart}\;\mathrm{disease}]\\ \; & +1[\mathrm{hypertension}]+1[\mathrm{BMI}>35]\\ \; & +1[\mathrm{cholesterol}=3]. \end{array}$$

where 1[·] denotes the indicator function of the corresponding clinical condition. The contributions of systolic blood pressure ($s_{\mathrm{SBP}}$) and glucose ($s_{\mathrm{glu}}$) are determined according to the following threshold rules:

(3) $$s_{\mathrm{SBP}}=\left\{\begin{array}{cc} 3, & \mathrm{if}\;\mathrm{SBP}\geq 180\;\mathrm{or}\;\mathrm{SBP}<80,\\ 2, & \mathrm{if}\;160\leq \mathrm{SBP}<180\;\mathrm{or}\;80\leq \mathrm{SBP}<90,\\ 1, & \mathrm{if}\;140\leq \mathrm{SBP}<160,\\ 0, & \mathrm{otherwise}. \end{array}\right.$$

(4) $$s_{\mathrm{glu}}=\left\{\begin{array}{cc} 2, & \mathrm{if}\;\mathrm{glucose}>300,\\ 1, & \mathrm{if}\;200<\mathrm{glucose}\leq 300,\\ 0, & \mathrm{otherwise}. \end{array}\right.$$

The final risk category is obtained by thresholding the composite score:

(5) $$y=\left\{\begin{array}{cc} 3\;\left(\mathrm{critical}\right), & s(x)\geq 7,\\ 2\;\left(\mathrm{high}\right), & 5\leq s(x)<7,\\ 1\;\left(\mathrm{moderate}\right), & 3\leq s(x)<5,\\ 0\;\left(\mathrm{low}\right), & s(x)<3. \end{array}\right.$$

The composite score is bounded between 0 and a theoretical maximum of 11, obtained by summing the maximum contribution of each term (3 for systolic blood pressure, 2 for glucose, 2 for stroke, and 1 each for heart disease, hypertension, BMI>35, and cholesterol=3), where the ordinal cholesterol level 3 denotes the well above normal (clinically high) category of the source dataset. The critical category (s(x)≥7) is therefore reached only by concurrence of several severe factors, which is consistent with the low observed prevalence of that class. The systolic-pressure bands in expression (3) intentionally follow the bidirectional MEWS logic, in which both marked hypotension (SBP<80) and marked hypertension (SBP≥180) are assigned the highest weight, because both represent acute physiological instability; the intermediate bands then grade increasing hypertensive severity. This banding is a deliberate simplification of the MEWS/Framingham rationale rather than a validated clinical instrument and is examined further in the Limitations.

The systolic-pressure, glucose, cholesterol, and body-mass contributions are provided by the primary Kaggle cohort (70,000 records [8]), whereas the binary comorbidity indicators for stroke (1[stroke]), heart disease (1[heartdisease]), and hypertension (1[hypertension]) originate from the Stroke Prediction (5110 records [9]) and Heart Failure Prediction (918 records [10]) cohorts. The three sources were concatenated into a single 76,028-record matrix (70,000+5110+918) over the union of their variables. For records in which a given indicator is structurally absent, the corresponding flag was set to its clinically negative value.

This deterministic framework constitutes the first analytical pathway, operating in parallel to the binary hybrid pipeline described below. This deterministic labeling strategy serves as the operational ground truth for model training and selection in the absence of longitudinal clinical outcome data. However, it is explicitly recognized that access to real observed outcomes, including hospital readmission, stroke events, and cardiovascular deterioration, would represent the most impactful next step toward the development of a truly prognostic model.

**Second Pathway:** To ensure scientific rigor and model generalizability, the training cohort (70,000 patient records [8]) was processed using physiological boundary filters to eliminate out-of-range inputs. Specifically, samples were restricted to systolic blood pressure (ap_hi∈[60,250]mmHg), height (height∈[1.00,2.20]m), and weight (weight∈[35,250]kg). We deliberately avoid the term “semi-supervised” for this pathway: the classifiers are trained in a fully supervised manner on the binary cardio outcome, and the unsupervised components (dimensionality reduction and K-Means) are used only for preprocessing and post hoc phenotype description. The pathway is therefore more precisely described as a *hybrid unsupervised-preprocessing plus supervised-classification* pipeline that sequentially integrates K-Nearest Neighbors (KNN) missing-data imputation, unsupervised dimensionality reduction/clustering, and supervised classification. This primary development cohort was used to train the predictive models and establish baseline risk architectures. An independent clinical cohort of 219 patients [10] was then used strictly as an external validation set. To clarify the earlier wording on “blinding”, the outcome labels of this external cohort were never presented to the models during training or threshold selection; they were revealed only at the final evaluation step, when predicted labels were compared against the held-out ground truth. Within the training cohort itself the cardio label is used as the supervised training target, as required for any supervised classifier. Both cohorts use the same *prevalent*-CVD definition of the outcome (documented presence or absence of disease, not future incident events); the external cohort is therefore an external *sample* of the same classification task rather than a prospective prognostic validation, and all discrimination results should be read in that light. The predictive engine comprises three supervised ensemble classifiers: Random Forest, Extreme Gradient Boosting (XGBoost), and Light Gradient Boosting Machine (LightGBM). The inclusion of both XGBoost and LightGBM is justified by their shared gradient boosting foundation combined with their complementary performance characteristics; XGBoost demonstrates greater robustness on smaller, well-curated datasets, while LightGBM offers superior computational efficiency when processing the larger combined dataset [12]. All implemented architectures were executed with a defined random state to guarantee reproducibility across both PCA and UMAP dimensionality reduction as well as supervised training methodologies. An overview can be found in Figure 1.

As a foundational phase prior to training pipeling and clustering, dimensionality reduction techniques, specifically Principal Component Analysis (PCA) and Uniform Manifold Approximation and Projection (UMAP), were applied to project the high-dimensional feature space into optimized, low-dimensional embeddings. Following the low-dimensional manifold transformations and the integration of insights from the trained supervised ensembles, centroid-based K-Means was executed within the reduced geometric space. This integrated process established definitive patient risk-group classifications and facilitated clinical phenotype mapping, ultimately uncovering latent sub-phenotypes and hidden patient topologies that elude traditional, rule-based diagnostic categories.

**K-Nearest Neighbors (KNN) filling:** In this study, the data preprocessing began with the use of K-Nearest Neighbors (KNN) algorithm, employed for data imputation to address missing values within a given dataset. Unlike simple statistical approaches, such as mean or median substitution, KNN estimates missing values by identifying the k most similar instances within the feature space. As the cardiovascular datasets contains incomplete patient records with missing critical indicators, KNN is utilized to predict these values. This method preserves the underlying data distribution, minimizing prediction error and ensuring the integrity of the clinical variables for subsequent analysis [14] without external information. The KNN algorithm determines the distance between the target observation (containing missing values) and all existing data points within the feature space; more information is available in the corresponding appendix.

This workflow is encapsulated within a reusable class designated as CVDDataPipeline. The architecture of this class ensures that the imputer’s training remains completely decoupled from any specific downstream process, thereby guaranteeing architectural consistency and reproducibility across every stage of the data analysis. Internally, the pipeline divides the clinical indicators into three distinct feature subsets based on their mathematical properties and measurement scales: continuous numerical variables, ordinal categorical data, and binary features. Specifically, the continuous subset encompasses age, height, weight, systolic blood pressure (ap_hi), and diastolic blood pressure (ap_lo). The ordinal subset includes semi-quantitative clinical indicators such as cholesterol and blood glucose levels (gluc). Finally, the binary subset captures lifestyle and physiological factors, including gender, smoke, alcohol consumption, and physical activity status. This distinction is of paramount importance, as the K-Nearest Neighbors (KNN) algorithm requires a uniform scale across all features before calculating spatial distances [15].

Consequently, a normalization procedure utilizing the MinMaxScaler is executed prior to imputation. This preprocessing step ensures that variables with wide numerical distributions, such as blood pressure or weight, do not artificially dominate the Euclidean distance calculations at the expense of lower-magnitude binary or ordinal features. Once the missing data points are successfully imputed, the scaling transformation is inverted to restore the clinical variables to their original physical ranges and preserving their diagnostic interpretability [15]. What distinguishes this approach from simple imputation techniques is the subsequent post-processing applied to the imputed categorical variables. Because the KNNImputer operates across a continuous mathematical domain, its calculated outputs for discrete features often result in fractional values that do not align with predefined clinical categories [15]. To resolve this discrepancy, each continuous estimate generated for a categorical or even ordinal variable is systematically mapped to its nearest valid discrete integer. This thresholding step ensures that the imputed data strictly adheres to real world clinical constraints, thereby preserving the logical integrity and coherence of the dataset. Following this foundational pipeline setup, the empirical outcomes concerning the synthetic missingness injection protocols, the corresponding validation criteria, the imputation performance metrics, and the selection of the optimal neighborhood hyperparameter k are comprehensively detailed in the Section 3.

**Data Normalization and Dimensionality Reduction:** During the initial preprocessing phase, the structural scales and underlying distributions of all continuous, binary, and integer variables were systematically verified across both datasets. Unit normalization was performed on baseline demographic attributes to ensure physical and biological interpretability; specifically, patient age was converted from days to years, and height was transformed from centimeters to meters. To optimize the data matrix and eliminate non-predictive administrative features, the unique patient identification (id) column was excluded from both datasets. Following exploratory analysis, the primary development cohort was partitioned into training (80%) and internal testing (20%) subsets using stratified random sampling to preserve the target class distribution. To prevent data leakage, feature scaling (StandardScaler) and dimensionality reduction techniques (Principal Component Analysis [PCA] and Uniform Manifold Approximation and Projection [UMAP]) were fitted strictly on $X_{\mathrm{train}}$. Subsequently, the parameters derived from $X_{\mathrm{train}}$ (e.g., feature means, standard deviations, and principal component eigenvectors) were applied to $X_{\mathrm{test}}$ and the independent external validation cohort exclusively via out-of-sample projection (transform operation). This ensured that both internal testing and external validation samples were projected onto the exact spatial coordinate system defined by the training manifold, avoiding geometric misalignment and providing an unbiased evaluation of predictive capacity.

Following feature standardization and KNN imputation, an optimization analysis was conducted to determine the ideal lower-dimensional projections for both the PCA and UMAP algorithms. For the Principal Component Analysis (PCA), the target dimensionality selection was constrained to capture a cumulative explained variance ratio within the 80% to 90% interval; this criterion was satisfied by selecting seven principal components. Concurrently, hyperparameter optimization for the Uniform Manifold Approximation and Projection (UMAP) framework determined that a 7-dimensional embedding space was optimal, where this projection achieved an exceptional structural trustworthiness score of 0.9944. The original index sequencing remained entirely unaltered keeping the original clinical tracking records and target diagnostic labels.

**Supervised training:** The supervised ensemble architectures were systematically trained and validated on these respective subsets. The Random Forest model relied on an architectural baseline of 250 discrete decision trees and the gradient boosting architectures were parameterized to balance training velocity with regularization constraints. LightGBM was initialized with 100 estimators, a learning rate (η) of 0.1, and a maximum tree depth constraint of 10. The XGBoost framework mirrored these foundational controls, utilizing 100 estimators and an identical learning rate of 0.05, but was intentionally restricted to a maximum tree depth of 6. From the training dataset, two partitions were taken: 80/20 for training and testing, denoted as X_train and X_test.

**Hyperparameter optimization and Threshold determination:** To maximize the predictive efficacy of the classification models, a systematic grid search cross-validation protocol was executed across the Random Forest, LightGBM, and XGBoost architectures. The optimization hyperparameter space for the ensemble size (n_estimators) evaluated the discrete array {100,150,200,250}, while the maximum tree depth constraint (max_depth) evaluated the structural boundaries defined by {6,8,10,12}. This procedure was executed with the training and testing partitions derived from the primary development cohort, which had been transformed via PCA and UMAP for dimensionality reduction. The optimization framework prioritized the area under the Receiver Operating Characteristic curve (roc_auc_score) as its primary performance metric. Given the binary classification target, high/low-risk groups, evaluating the precision of the continuous probability assignments was the most important feature to analyze. In cardiovascular diagnostics, the capacity to correctly identify true-positive instances while minimizing false negatives, wherein critically ill patients are dangerously misclassified as healthy, carries far greater clinical significance than optimizing global accuracy. To establish a clinically safe diagnostic boundary rather than relying on the arbitrary 0.50 default threshold, Youden’s *J* statistic was leveraged to mathematically pinpoint the optimal geometric cutoff coordinate on the ROC curve.

**Validation testing:** The final trained supervised models were evaluated on the external validation cohort (n=219 patients [10]). For each patient vector, the model calculated the predicted probability of high-risk classification. Youden’s *J* statistic was then applied to establish the optimal cutoff for assigning patients to either the high-risk (1) or low-risk (0) group. The Youden’s *J* cutoff was determined on the [training / internal validation] partition and then applied unchanged to the external cohort, so that threshold selection did not rely on the evaluation data. Finally, these predicted labels were compared against ground-truth cardiovascular disease outcomes to evaluate model performance and quantify correct patient classifications.

**Clustering algorithms:** A parallel unsupervised learning pipeline was established to address the interpretability constraints inherent the "black-box" machine learning paradigms. While the integrated dimensionality reduction and ensemble classification frameworks excel at optimizing posterior risk probability mapping, they often obscure the underlying data structures, rendering direct clinical interrogation difficult. To bridge this gap and establish absolute clinical transparency, a partition-based K-Means clustering algorithm was deployed on the training cohort and the validation one. The explicit objective upon this algorithm was the data-driven discovery of distinct clinical phenotypes rather than class optimization. By mapping the training cohort the K-Means algorithm identified stable patient subgroups and derived their characteristic parameter centroids, determining core feature values across defined cardiovascular risk groups. This clustering enables a transparent breakdown of the exact clinical intervals, biomarker concentrations, and lifestyle indicators that characterize both low- and high-risk cardiovascular cohorts following supervised model outputs, providing an interpretable reference matrix for clinical validation. Importantly, the outcome variable (cardio) and the comorbidity indicators were *not* included in the feature matrix used for clustering; cardio is appended only after cluster assignment, as a post hoc descriptor, so that any association between a cluster and disease status reflects only a way of comparison.

## 3. Results

This section presents the experimental results obtained from the two methodological pathways proposed for cardiovascular disease (CVD) risk assessment. The first pathway evaluates supervised risk stratification models trained on a multi-cohort dataset, whereas the second pathway validates the binary hybrid pipeline, including KNN imputation, dimensionality reduction, ensemble classification, and unsupervised phenotype discovery.

### 3.1. Rule-Learnability Verification (Pathway I)

#### 3.1.1. Risk Group Distribution

The four-level clinical risk label was generated using the deterministic scoring strategy described in the Section 2. The resulting distribution revealed a strongly imbalanced cohort structure: 73.2% of patients were categorized as low risk, 22.2% as moderate risk, 4.3% as high risk, and only 0.26% as critical risk.

This pronounced asymmetry demonstrates that conventional accuracy metrics are insufficient for reliable model comparison, as a trivial majority-class classifier would achieve high overall accuracy while failing to identify clinically relevant high-risk patients. Consequently, the macro F1-score was selected as the primary evaluation metric, and SMOTE oversampling was applied exclusively to the training partition. The train/validation/test split was analyzed independently within each cross-validation fold, so that no synthetic sample derived from a held-out record could leak into evaluation.

Because the comorbidity indicators for stroke, heart disease, and hypertension are present only in the Stroke Prediction and Heart Failure cohorts (6028 records) and are set to their clinically negative value for the 70,000 Kaggle records, the terms 2·1[stroke]+1[heartdisease]+1[hypertension] are non-zero almost exclusively for records originating from the two smaller cohorts. These terms therefore behave, in part, as an indicator of data provenance, and the four-level label *y* partly encodes cohort of origin rather than purely clinical risk. Crucially, it does not affect the study’s prognostic claims: Pathway I is reported only as a rule-recovery consistency check (Section 4), and all out-of-sample discrimination claims rest on Pathway II, which is trained and validated entirely within the single self-contained Kaggle cohort family and never uses the pooled four-level label.

#### 3.1.2. Comparison of Classification Models

Nine supervised classification architectures were evaluated under identical preprocessing and validation conditions using the unified 76,028-record dataset [8,9,10]. The unified cohort was partitioned into training, validation, and test subsets using a [70/15/15] stratified split that preserved the four-level class distribution; SMOTE was applied exclusively to the training partition, and all validation and test metrics reported below were computed on data unseen during fitting. Table 1 summarizes the comparative performance obtained on the validation and test partitions.

Among all evaluated architectures, LightGBM achieved the highest validation macro F1-score (0.9870), the best cross-validation F1-score (0.9954), and a test ROC-AUC of 0.9999. Random Forest also demonstrated strong performance, whereas Logistic Regression and k-NN produced substantially lower macro F1-scores, indicating limited capability to model the non-linear interactions present in the clinical feature space.

#### 3.1.3. Selected Model Performance

The selected LightGBM model achieved near-perfect discrimination for the low- and moderate-risk strata, with precision and recall values exceeding 0.995. Performance remained clinically acceptable for the high-risk (3305) and critical-risk (196) categories despite their limited representation in the original cohort.

The critical-risk class contained only 196 records in the original dataset, which explains the slight reduction in recall observed on the final holdout test partition. Nevertheless, the model maintained strong discriminative performance across all four risk levels, supporting its use as the final classifier for the first pathway.

### 3.2. Binary Hybrid Pipeline Learning (Pathway II)

#### 3.2.1. KNN Imputation Validation

The binary hybrid pathway began with the validation of the KNN imputation strategy. Artificial missing values were introduced into a complete reference subset of n=4000 records drawn from the training partition, using corruption rates of 15% for numerical variables and 10% for categorical variables, yielding a total of 4800 masked cells (12.0% of the complete feature matrix). Because this subset is drawn from the training data, the reconstruction metrics reported below should be read as an internal (potentially optimistic) estimate of imputation quality rather than an out-of-sample one. We also note that two variables (age and cholesterol) did not meet the acceptance thresholds; since the phenotype clustering (Section 3.3) is performed on imputed data, part of the residual variation between clusters may reflect imputation error rather than genuine clinical structure. This is a further reason to treat the clustering as descriptive, and a complete-case (imputation-free) re-analysis is identified as future work.

The distribution of masked entries across all variables is presented in Figure 2. A supervised validation procedure was then performed by comparing imputed values against the original ground-truth observations.

#### 3.2.2. Selection of the Optimal k

Candidate neighbor values k∈{3,5,7,10} were evaluated using relative RMSE for numerical variables and classification accuracy for categorical variables. Figure 3 summarizes the validation results.

The optimal configuration was obtained with k=3, which simultaneously achieved the lowest mean relative RMSE (≈11.66%) and the highest mean categorical accuracy (≈84.4%) among the evaluated neighborhood sizes. Larger neighbor values progressively increased the reconstruction error and, in general, reduced categorical accuracy, indicating that additional neighbors introduced excessive smoothing without improving reconstruction quality.

#### 3.2.3. Per-Variable Imputation Performance

Figure 4 presents the reconstruction performance obtained with the selected k=3 configuration.

Eight of the eleven clinical variables, excluding diastolic blood pressure which was not included in this validation, satisfied the predefined acceptance criteria. Only *age* (relative RMSE 21.36%) and *cholesterol* (imputation accuracy 0.700) fell outside the acceptance thresholds, showing interpretable limitations associated with the wide numerical range of age and the low-cardinality categorical distribution of cholesterol.

#### 3.2.4. Dimensionality Reduction

Both PCA and UMAP were evaluated to determine the optimal latent base representation of the training dataset. For PCA, the cumulative explained variance criterion indicated that seven principal components preserved the target information range, as shown in Figure 5.

For UMAP, trustworthiness analysis (0.9944) identified a seven-dimensional embedding too as the most stable representation, Figure 6.

#### 3.2.5. Ensemble Classifier Comparison

The PCA and UMAP feature representations were combined with Random Forest, LightGBM, and XGBoost classifiers. Table 2 summarizes the resulting performance.

Based on initial inspection of Table 2, XGB+PCA appears to be the leading model as it achieved the highest ROC-AUC score. However, these results must be evaluated on the validation cohort to establish a final assessment, which is substantially smaller than the primary dataset, exhibits less noise, and shares the same variable categorization.

All external-cohort results are reported with measures of uncertainty. For each of the six Pathway II pipelines evaluated on the 219-patient cohort we report accuracy with a 95% bootstrap confidence interval (3000 resamples), together with sensitivity, specificity, positive and negative predictive values, balanced accuracy, F1-score, the area under the ROC curve computed on the external cohort, and the Brier score. Because all pipelines are evaluated on the same patients, differences between UMAP- and PCA-based variants of each classifier were assessed with the paired McNemar test (exact binomial form on the discordant pairs); a two-sided p<0.05 was considered significant. For the unsupervised analysis, the number of clusters was examined with the elbow method complemented by the silhouette coefficient over k∈{2,…,8}, and clustering stability was quantified by the adjusted Rand index (ARI) across bootstrap resamples. To test whether the clusters encode the outcome beyond chance, the association between cluster assignment and cardio was measured with the normalized mutual information and Cramér’s *V* and compared against a null distribution obtained by permuting the cardio labels 2000 times; the complete preprocessing chain (interval filtering, scaling, imputation) was additionally audited to confirm that no step depends on the outcome. Analyses were performed in Python; the exact library versions and random seeds are reported in the Data Availability statement and the public repository.

Across the six pipelines the external-cohort accuracy ranged narrowly from 70.3% to 74.4% (a spread of nine patients), and the 95% confidence intervals overlap almost completely, indicating no statistically meaningful separation among configurations. To formally compare the UMAP- and PCA-based variants of each classifier on the same 219 patients, we applied paired McNemar tests: LightGBM (p=0.63), XGBoost (p=0.52), and Random Forest (p=0.80) all showed no significant difference. We therefore do *not* claim that UMAP outperforms PCA, and we do *not* select any of the six pipelines as superior; all six are reported as equivalent on this cohort. Where a single configuration is shown for illustration (e.g. in the probability and ROC figures), this choice is for presentation only and does not imply better performance. The external-cohort AUC (0.82–0.85) is reported here for the first time as a same-set discrimination measure, complementing the training-set AUC of Table 2. A post hoc power analysis makes the limitation explicit: with n=219 and a discordant-pair proportion of ∼8–10% (as observed), the paired McNemar test had only ≈46% power to detect even a four-percentage-point accuracy difference; reaching 80% power for a four-point difference would require roughly n≈ 500 patients, and detecting the differences actually observed (≤2 points) would require several thousand. The external cohort is thus far too small to distinguish the pipelines, which is why none is preferred.

#### 3.2.6. Posterior Probability Distributions

Figure 7 compares the posterior probability distributions generated by the six integrated architectures.

Whereas PCA-based models yielded a clearer bimodal separation between low-risk and high-risk patients, UMAP-based models concentrated a larger proportion of cases around the intermediate probability region. This concentration in the uncertain region is consistent with slightly poorer probability calibration for the UMAP variants (reflected in marginally higher Brier scores in Table 3) and should be interpreted as a calibration difference rather than as evidence of better discrimination.

#### 3.2.7. Probability Concordance Analysis

The agreement between PCA- and UMAP-based probability assignments was evaluated at the patient level using the validation cohort.

As shown in Figure 8, there are no substantial discrepancies between models. As indicated in Table 3, differences in the number of hits and misses remain minimal, demonstrating a high degree of concordance in probability assignments across both PCA- and UMAP-based models.

#### 3.2.8. ROC Curve Analysis

The ROC curves obtained for the six integrated architectures are presented in Figure 9.

While the PCA-based XGBoost and Random Forest models achieved the highest discriminative performance on the training cohort based on ROC-AUC metrics, the UMAP-based pipelines (LightGBM, XGBoost, and Random Forest) notably yielded the highest number of correct classifications on the validation cohort. ROC-AUC values remained strong across models at approximately 0.79, with the highest Youden’s cutoff observed in the Random Forest + UMAP pair and the lowest in the XGBoost + UMAP architecture, based on training cohort.

### 3.3. Unsupervised Phenotype Discovery

#### 3.3.1. Selection of the Number of Clusters

K-Means clustering was applied to the training partition ($X_{\mathrm{train}}$), the training dataset, to identify latent clinical phenotypes for that dataset only. After interval-based filtering (which retained 69,724 records from the primary cohort) and the stratified 80/20 split, clustering was performed on the 55,779 training patients. The elbow method indicated an optimal configuration of five clusters.

The inflection point observed at k=5 in Figure 10, was selected as the final clustering configuration because additional clusters produced only marginal reductions in within-cluster inertia.

#### 3.3.2. Phenotype Characterization

Table 4 summarizes the centroid values of the resulting clinical phenotypes evaluated in training cohort.

Table 5 summarizes the centroid values of the resulting clinical phenotypes evaluated in the validation cohort.

#### 3.3.3. Clinical Interpretation of Phenotypes

The identified phenotypes revealed distinct combinations of metabolic, demographic, and lifestyle characteristics. Elevated cholesterol and glucose levels were the principal variables differentiating the higher risk phenotype from the lower risk groups, whereas diastolic blood pressure remained comparatively stable across clusters. The algorithm clustered only on the ten standardized input features (age, sex, height, weight, systolic pressure, cholesterol, glucose, smoking, alcohol, physical activity), and cardio is reported solely as a post hoc descriptor.

Importantly, and in contrast to what the modal (0/1) values of the previous version suggested, the clusters do *not* separate patients cleanly by disease status. The per-cluster CVD prevalence in the training cohort ranges only from 0.468 to 0.651 around an overall prevalence of 0.500; the earlier table reported the modal class of the binary outcome, which for a binary variable is always exactly 0 or 1 and therefore created a spurious appearance of perfect 100%/0% separation (for example, training Phenotype 3 in the validation cohort is an exact 11/11 split, i.e., a 50% prevalence displayed as 0). We have replaced those modal entries with the true prevalence and raw counts (Table 4 and Table 5). The association between cluster assignment and cardio is correspondingly weak: the normalized mutual information is 0.005 in the training cohort and 0.054 in the validation cohort (Cramér’s V=0.11 and 0.32). A label-permutation test (2000 shuffles of cardio) confirms that these associations, although statistically greater than chance (p<0.001), are far below the value that outcome leakage would produce (a leaked outcome would drive the mutual information towards its maximum of 1). This directly resolves the apparent tension with the supervised results: because the clusters carry only a small amount of information about the outcome, it is fully expected that the supervised classifiers, which are optimized for the task, reach 70–74% accuracy while the unsupervised partition is only mildly enriched for disease. As an additional check, the entire preprocessing chain was audited for leakage: the interval-based filtering uses only physiological bounds on ap_hi, height and weight (never cardio), the StandardScaler is fitted on the training partition alone and applied to held-out data by transform, and KNN imputation is not part of the clustering branch. Taken together, these analyses found no evidence of outcome leakage in the unsupervised branch; because a low mutual information is still compatible with a weak or partial leakage effect, this residual possibility cannot be excluded and is acknowledged in the Limitations. Cluster validity was quantified with the silhouette coefficient, which peaked near k=5–6 (training cohort), and with a bootstrap adjusted Rand index of ≈0.68 at k=5; the subgroups are therefore reproducible but descriptive rather than sharply separated.

The permutation test summarized in Table 6 makes this quantitative: for both cohorts the observed association lies far in the upper tail of the permutation null yet remains very small in absolute terms, two to three orders of magnitude below the value (NMI→1) that outcome leakage would produce.

The validation cohort was clustered with an *independent* K-means fit on the 219 external patients. Its clusters are therefore not the same groups as the training phenotypes and cannot be compared with Table 4 cluster by cluster; to make this explicit, they are labeled V-cluster 1–5 (Table 5). Four of the five clusters have a CVD prevalence close to the cohort average (0.43–0.50), and only V-cluster 5 is markedly enriched, with a prevalence of 0.906 (29/32; Wilson 95% confidence interval [0.758,0.968]). This estimate rests on only 32 patients and must be read with caution; the cluster does not contain all disease cases, since the 114 positive patients are distributed across all five clusters. Because several of these validation clusters are small (as few as 22 patients), the corresponding mean profiles are reported as descriptive observations only and are not the basis for statistical claims. V-cluster 5 was characterized by the highest cholesterol level (3.0), Stage 2 systolic hypertension (141.97mmHg), and the highest mean body weight (79.72kg), a profile that is clinically coherent with elevated—though not deterministic—cardiovascular risk.

## 4. Discussion

The present study proposes a dual-pathway machine learning framework for cardiovascular disease (CVD) risk stratification that integrates supervised prediction, a hybrid learning pipeline, dimensionality reduction, and unsupervised phenotype discovery. Unlike conventional binary classification approaches, the proposed methodology combines clinically interpretable risk categorization with probabilistic prediction and phenotype characterization, providing complementary information that may support clinical decision-making in resource-constrained environments.

### 4.1. Performance of the Four-Level Risk Stratification Model

The first methodological pathway demonstrated that gradient boosting algorithms consistently outperformed linear and distance-based classifiers for cardiovascular risk prediction. Among the nine evaluated architectures, LightGBM achieved the highest macro F1-score and ROC-AUC while maintaining robust performance across all four risk categories.

These metrics must be interpreted with care. Because the four-level label *y* is generated as a deterministic function of the same clinical variables subsequently used as model inputs (systolic blood pressure, glucose, cholesterol, body mass index, and the binary comorbidity indicators), this benchmark does not quantify clinical predictive capacity in a prognostic sense; rather, it measures the fidelity with which each classifier recovers a known closed-form thresholding rule. The near-perfect scores should therefore be read as a rule-recovery consistency check, confirming that the scoring rule is learnable and directly deployable without re-implementing it, rather than as evidence of diagnostic performance. Genuine out-of-sample discrimination against an independent clinical ground truth is reported in the binary hybrid pipeline, where the ROC-AUC settles around 0.79.

The superior performance of LightGBM is consistent with previous studies reporting the advantages of gradient boosting methods for structured clinical datasets [6,12,16]. Cardiovascular risk is influenced by multiple non-linear interactions among demographic characteristics, blood pressure measurements, metabolic variables, and lifestyle factors. These relationships cannot be adequately represented by linear decision boundaries, explaining the comparatively poor performance observed for Logistic Regression and k-nearest neighbors [7,13].

An important characteristic of the proposed model is the adoption of macro F1-score as the primary optimization criterion. Because only 0.26% of the complete cohort belonged to the critical-risk category, conventional accuracy would provide an overly optimistic estimate of predictive performance. By equally weighting all classes, macro F1-score provides a more clinically meaningful assessment for highly imbalanced medical datasets.

### 4.2. Binary Hybrid Learning Pipeline

The second pathway demonstrates that combining missing-value imputation, dimensionality reduction, and supervised learning provides a robust framework for cardiovascular risk estimation in partially incomplete datasets. Artificially masking observations enabled an objective evaluation of the KNN imputation strategy, where k=3 achieved the best compromise between reconstruction error and categorical accuracy.

Although two variables exceeded the predefined acceptance thresholds, these deviations can be explained by their intrinsic statistical properties. Age naturally presents a wide numerical range, increasing reconstruction error, whereas cholesterol contains relatively few categorical levels, making it more susceptible to ambiguity during distance-based imputation. Despite these limitations, the overall reconstruction accuracy remained acceptable for subsequent predictive modeling.

These findings suggest that KNN imputation constitutes an effective preprocessing strategy for clinical datasets where missing observations are unavoidable, particularly when combined with careful hyperparameter optimization [15,17].

### 4.3. PCA Versus UMAP for Clinical Prediction

A central methodological point of this study is that PCA and UMAP are statistically indistinguishable for risk group classification task. On the training cohort PCA yielded marginally higher ROC-AUC, and on the external cohort UMAP yielded marginally more correct classifications, but both differences are within noise: the external accuracies span only 70.3–74.4% with fully overlapping confidence intervals, and paired McNemar tests between the UMAP and PCA variants of every classifier were non-significant (LightGBM p=0.63, XGBoost p=0.52, Random Forest p=0.80). Comparing a training set AUC against a validation set hit count, as an earlier reading of the results did, is not a valid basis for preferring one reduction over the other, because the two quantities are measured on different data and capture different aspects of performance (probability ranking versus post-threshold classification). We therefore refrain from claiming any superiority of UMAP over PCA.

A further consideration favours caution specifically for UMAP. Unlike PCA, whose .transform() is an exact linear projection onto fixed eigenvectors, UMAP was not designed for out-of-sample embedding: its .transform() produces an *approximate* placement of new points onto a previously learned manifold, which is a known limitation for tabular clinical data and adds an additional, hard-to-quantify source of variability to the external-cohort results. This reinforces our decision not to over-interpret small UMAP–PCA differences. The practical implication is that, for structured clinical data of this dimensionality, the choice between PCA and UMAP can be made on secondary grounds (interpretability, in terms of computational cost, PCA-driven methods were faster than UMAP-based ones, robustness of out-of-sample projection) rather than on a predictive-performance advantage that the data do not support.

### 4.4. Clinical Interpretation of the Identified Phenotypes

The incorporation of unsupervised learning provides an additional layer of interpretability beyond traditional prediction models. Rather than focusing exclusively on classification accuracy, K-Means clustering identified clinically meaningful patient phenotypes characterized by distinct combinations of demographic, metabolic, and lifestyle variables.

Interestingly, age and height remained relatively stable across the identified phenotypes, whereas cholesterol and glucose levels emerged as the primary sources of cluster separation. These findings agree with current evidence identifying metabolic dysfunction as one of the strongest predictors of cardiovascular disease progression [18].

Read strictly against the tables, the phenotypes should be interpreted as descriptive, hypothesis-generating groupings rather than validated clinical phenotypes, and sex is not a driver of risk: for example, training Phenotypes 2 and 3 share the same modal sex yet have markedly different CVD prevalence (0.65 vs. 0.47), so the higher prevalence of Phenotype 2 tracks its elevated cholesterol (level 3) and glucose (level 3) rather than sex. Consistently, the most enriched validation cluster (V-cluster 5) is characterized by the highest cholesterol level (3.0), the highest systolic blood pressure and the highest mean weight, but not by elevated glucose. Taken together, the clustering points to metabolic variables (cholesterol, and to a lesser extent glucose and adiposity) as the main axes of separation, whereas physical activity, alcohol intake and smoking do not clearly distinguish the higher-prevalence groups. These observations are associations within a single dataset and are not offered as clinical phenotype discovery.

The ability to identify clinically interpretable patient phenotypes may facilitate personalized preventive strategies by allowing healthcare professionals to recognize characteristic combinations of risk factors rather than evaluating isolated variables independently.

### 4.5. Clinical Implications

From a clinical perspective, the proposed dual-pathway framework offers several practical advantages. First, the four-level risk stratification provides substantially richer information than conventional binary classification, allowing healthcare professionals to prioritize interventions according to disease severity.

Second, the probabilistic outputs generated by the supervised models may facilitate individualized risk communication while preserving model interpretability. Finally, the unsupervised phenotype analysis offers complementary information that may improve patient profiling and support precision medicine initiatives.

Because all models were developed using routinely collected clinical variables, the proposed framework could be implemented in primary healthcare settings without requiring additional laboratory or imaging resources, making it particularly attractive for low-resource environments [2,7].

### 4.6. Limitations

Several limitations should be acknowledged and are consolidated here.

*Nature of the Pathway I label:* The four-level label is derived deterministically from the input variables, so the near-perfect scores of Section 3.1 reflect faithful recovery of the scoring rule rather than independent prognostic validity. Pathway I should be read only as evidence that the clinical rule is learnable and deployable, not as a diagnostic model.

*Label cohort confounding:* As detailed in Section 3.1.1, the stroke, heart disease, and hypertension terms are populated only from the two smaller cohorts and are otherwise set to zero, so the pooled four-level label partly encodes data provenance. A source-stratified label distribution and a single-cohort sensitivity analysis (Kaggle-only, using the commonly available variables) are the priority confirmatory analyses; the prognostic claims of this work rest on Pathway II, which does not use the pooled label.

*Clinical target and absence of real outcomes:* Pathway II classifies *prevalent* cardiovascular disease (the documented cardio status), not incident future events; claims are framed accordingly, and no prognostic or time-to-event interpretation is made. Incorporating longitudinal outcomes (readmission, stroke, cardiovascular deterioration) is the most direct route to genuine prognostic validity.

*External sample size:* The external cohort comprises only 219 patients; differences among pipelines amount to a handful of cases, are not statistically significant (paired McNemar, all p>0.5), and require a substantially larger cohort to confirm.

*Out-of-sample UMAP projection:* UMAP’s .transform() yields only an approximate embedding of new points, an intrinsic limitation for external validation on tabular data that adds variability not present in the exact PCA projection.

*Data source and generalizability:* Both cohorts are public, single-source datasets rather than prospective clinical studies, which may introduce selection bias and limit population representativeness.

*Validation-cohort phenotypes:* The phenotypes in the validation cohort were obtained from a separate K-means fit rather than by projecting patients onto the training centroids, so the two phenotype tables are not matched cluster by cluster and are labeled independently. In addition, some validation clusters are small (down to 22 patients, and the enriched cluster rests on 32), so their profiles are descriptive only. Finally, although the preprocessing audit and the permutation test found no evidence of outcome leakage in the unsupervised branch, a low mutual information cannot by itself exclude a weak or partial leakage effect.

*Class imbalance:* Despite SMOTE on the training partition, the critical-risk stratum remained rare, so uncertainty for that group is higher than for the others; results without SMOTE and the pre/post-resampling class distribution are reported as a control.

Overall, the framework should be regarded as a reproducible, interpretable methodological contribution whose clinical utility must be confirmed through prospective, validation using longitudinal outcomes before routine implementation, as well as in situ population controls with available cardiovascular clinical histories.

## 5. Conclusions and Future Work

This study presented a two-pathway machine learning framework for cardiovascular risk stratification whose main contribution is Pathway II, a hybrid unsupervised-preprocessing plus supervised-classification pipeline. Pathway I served only to verify that the deterministic four-level rule is learnable; its near-perfect recovery (e.g. validation macro F1-score =0.9870) is a consistency check, not predictive evidence, and is not a contribution of this work. In Pathway II, evaluated on the independent 219-patient cohort, the six ensemble–reduction pipelines performed comparably (accuracy 70–74%, external-cohort AUC 0.82–0.85), and paired McNemar tests found no significant difference between UMAP- and PCA-based variants of any classifier (all p>0.5). We therefore report the framework as a reproducible, interpretable methodology and explicitly refrain from claiming superiority of either dimensionality-reduction technique or genuine prognostic ability on the present data. In Pathway II, evaluated on the independent 219-patient cohort, the six ensemble reduction pipelines performed comparably (accuracy 70–74%, external-cohort AUC 0.82–0.85), and paired McNemar tests found no significant difference between UMAP- and PCA-based variants of any classifier (all p>0.5). We therefore report the framework as a reproducible, interpretable methodology and explicitly refrain from claiming superiority of either dimensionality-reduction technique or genuine prognostic ability on the present data.

Several limitations should be acknowledged. The four-level risk label relies on a deterministic scoring rule rather than longitudinal clinical outcomes, and its composite thresholds may not generalize directly to other demographic or clinical settings. The severe class imbalance in the critical-risk stratum (196 records, 0.26% of the combined cohort) constrained model performance for this category. Additionally, the 219-patient external validation cohort, while independent, remains modest in size relative to the primary development cohort. Incorporating real longitudinal outcome data, including hospital readmission, stroke events, and documented cardiovascular deterioration, represents the most direct path toward strengthening the prognostic validity of both pathways. The architectures of the PCA- and UMAP-based models showed no differentiating metrics due to the class characteristics of risk classification in the validation group and the similar metrics in the training phase. We highlight only a small number of hits and misses in small datasets.

Beyond the scope of this study, the validated pipeline is currently being explored as the predictive foundation for a home-based diagnostic application intended to extend cardiovascular risk stratification into non-clinical settings. This direction remains in early development and has not yet undergone field validation; it introduces additional considerations not addressed here, including on-device inference constraints, patient-facing interpretability of risk alerts, and validation against real world, self-reported physiological measurements rather than clinically curated cohorts. Future work will report on the design and evaluation of this application once such validation is complete.

A concrete implementation pathway is being specified within the Minciencias-funded home-monitoring project (contract 578-2022) from which the datasets and clinical motivation of this study arise. In that consumer-facing setting, the input features are not read from a curated clinical table but gathered through a four-layer edge-to-cloud architecture. Static and self-reported variables (age, sex, known comorbidities, and the cholesterol and glucose categories) are entered once at enrollment through a role-based user-management module whose interface is adapted for older adults, whereas the dynamic physiological variables are acquired continuously and non-invasively by low-cost wearables (heart rate, daily active-walking minutes and distance) and by an inertial unit (a triaxial accelerometer/gyroscope for mobility and posture), complemented by a timed electronic pill dispenser that timestamps medication adherence. Sensors stream to the patient’s or caregiver’s smartphone over Bluetooth Low Energy, and the phone acts as a gateway that forwards data to the cloud over Wi-Fi or cellular (3G/LTE) links according to local coverage.

Two design choices directly address the edge-computing and data-quality constraints raised in review. First, because connectivity in the target regions is intermittent, the mobile application maintains a local cache and performs automatic chronological store-and-forward synchronization once the network is restored, so that transient outages produce recoverable gaps rather than lost records. Second, those gaps, together with the noisier and partially self-reported consumer inputs, are precisely the missing-data regime that motivated the KNN-imputation and dimensionality-reduction stages evaluated in this work: the same preprocessing is intended to reconstruct incomplete feature vectors before inference, while a hybrid alert engine that couples physician-personalized deterministic thresholds with machine learning models issues notifications only for genuine anomalies, mitigating alarm fatigue. Heavier analytics remain in the cloud layer, keeping on-device requirements minimal. A dedicated study will report the prospective validation of this deployment, including calibration against sensor-derived rather than registry-curated measurements, which we regard as a prerequisite before any clinical use.

## Figures and Tables

**Figure 1:**
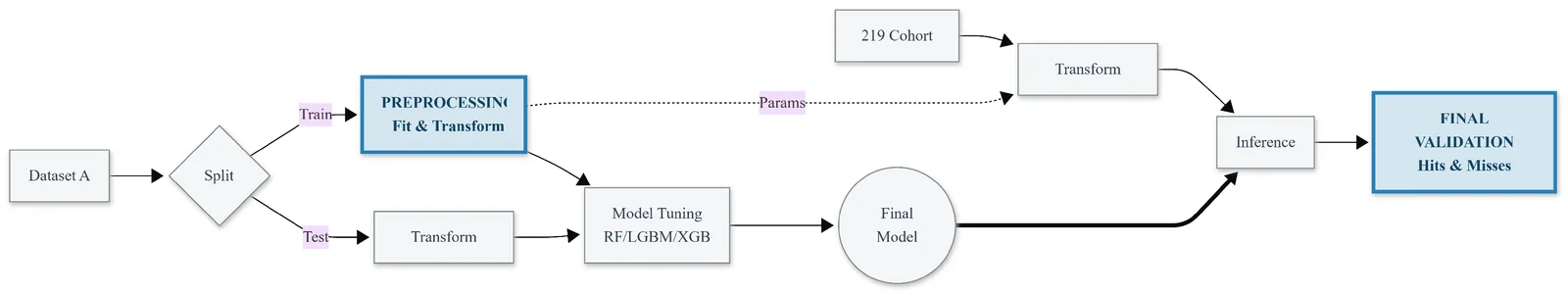
Architectural workflow schematic emphasizing the integrated dimensionality reduction and supervised ensemble training procedures. Panels details the primary derivation methodology executed on the training cohort, including feature compression and grid search optimization and also included the deployment phase using the independent validation cohort to compute individual posterior risk probabilities for final high-risk cardiovascular group classification.

**Figure 2:**
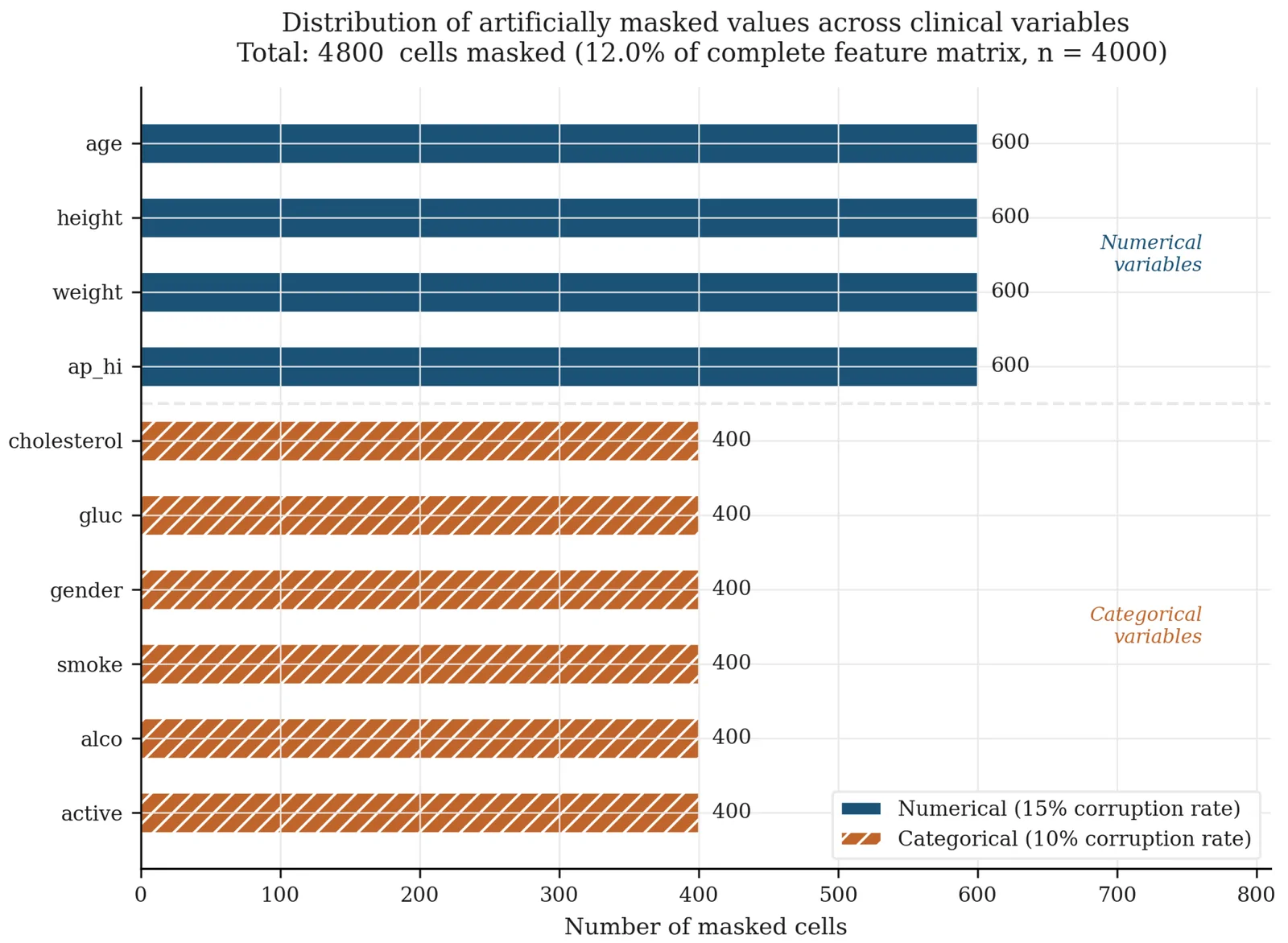
Distribution of artificially masked values across the clinical variables of the development cohort.

**Figure 3:**
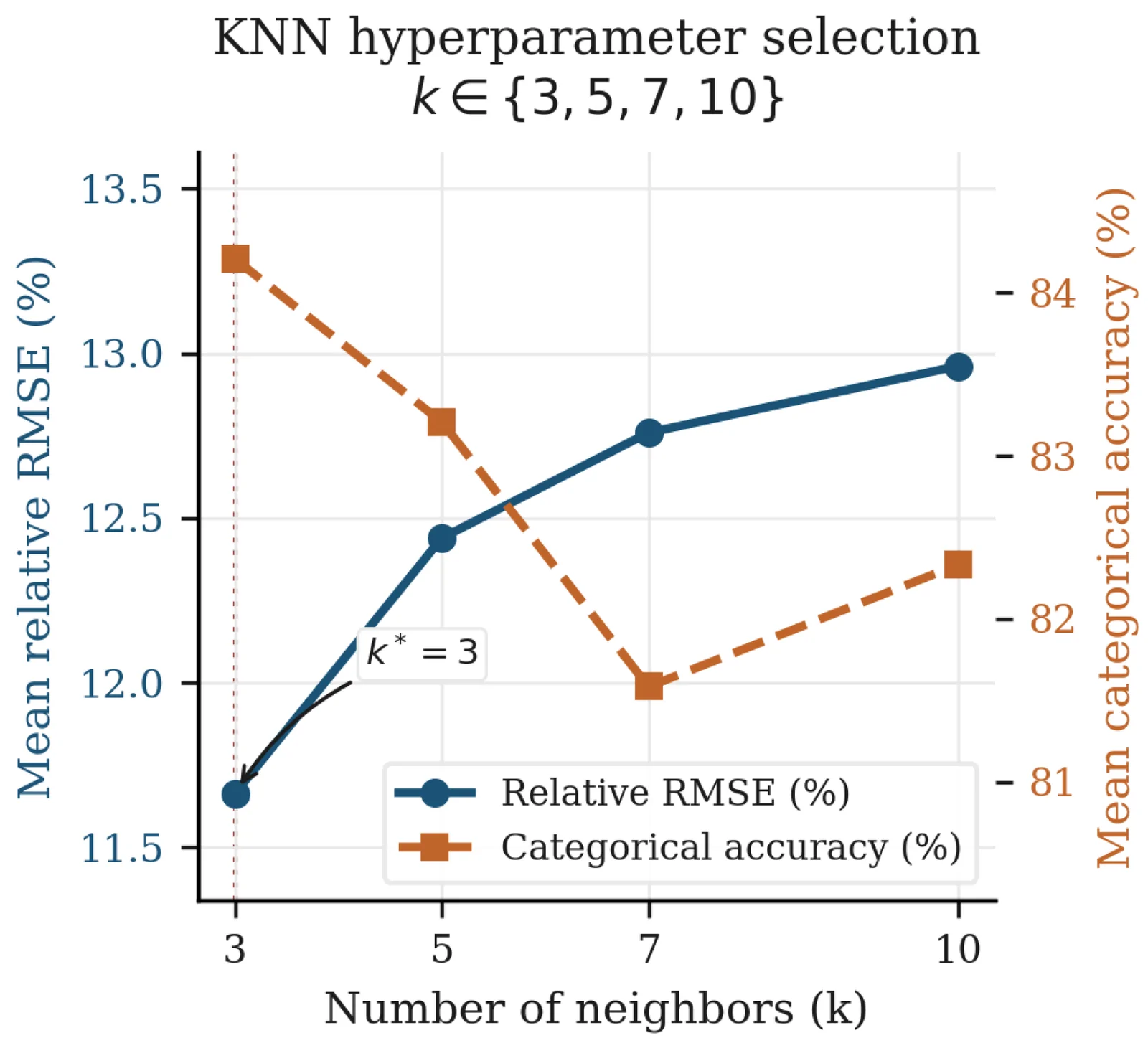
Hyperparameter selection for the KNN imputation pipeline. From the neighboors set k∈3,5,7,10, three was selected as the optimal, denoted by $k^{*}$.

**Figure 4:**
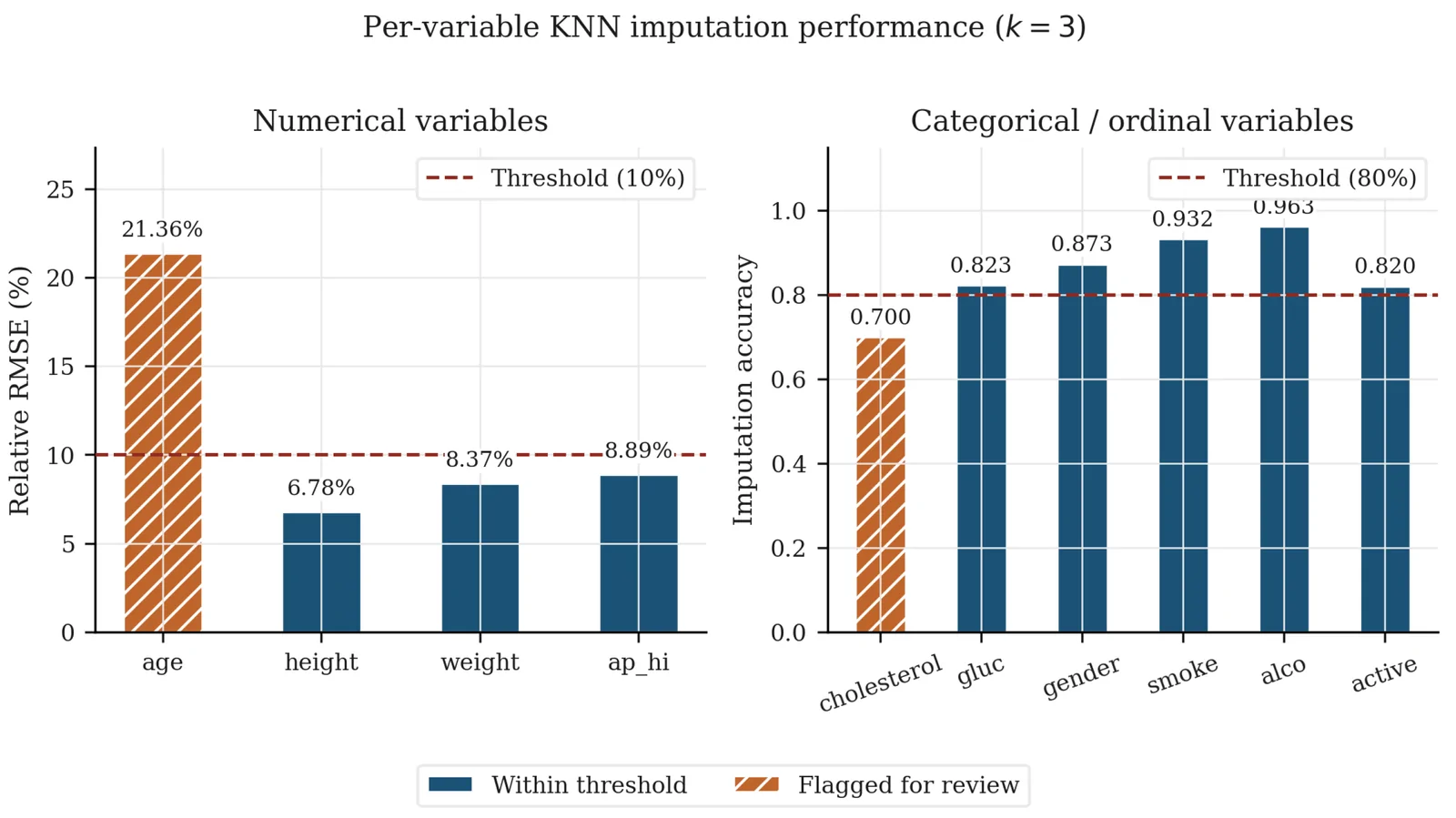
Per-variable KNN imputation performance with k=3.

**Figure 5:**
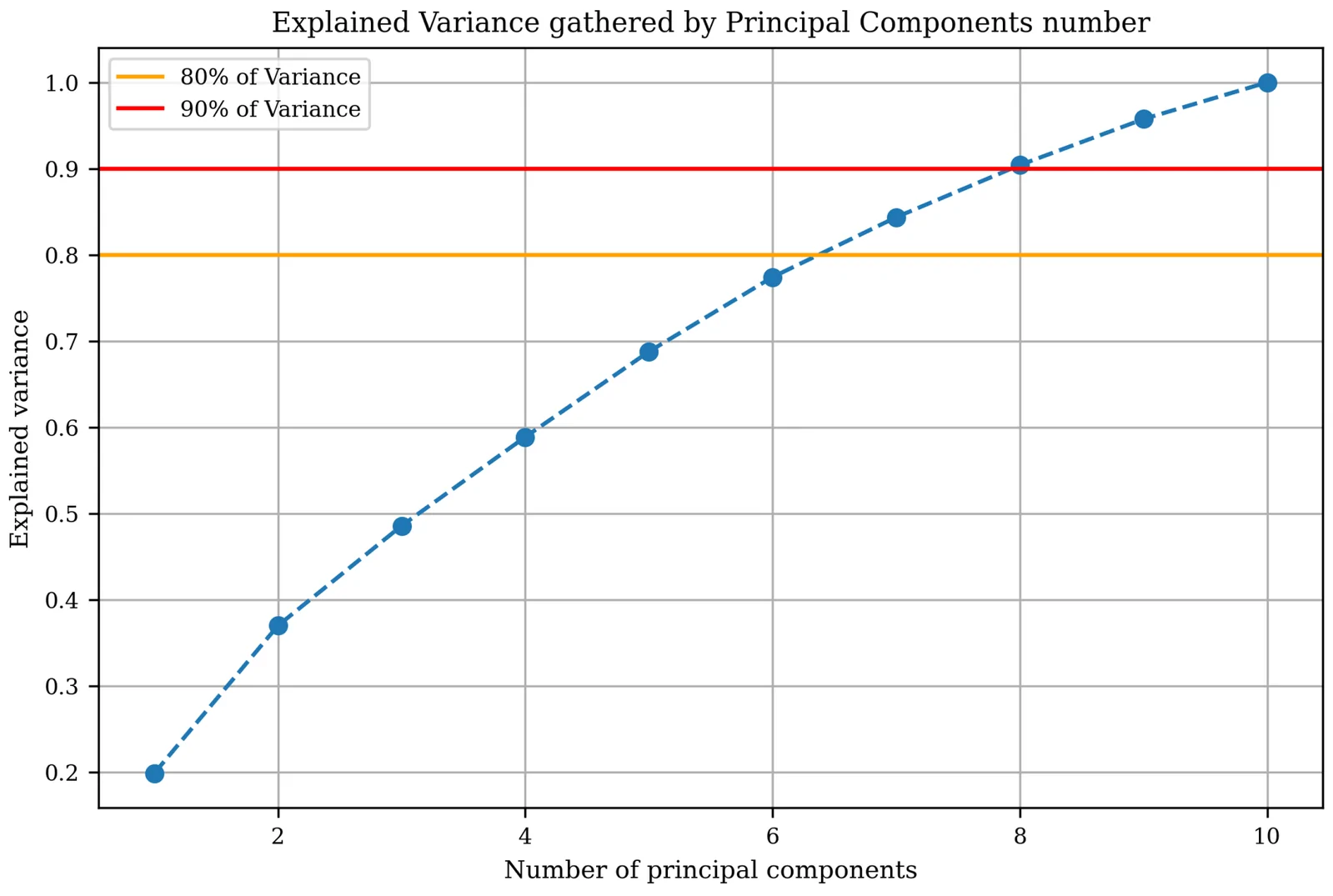
Selection of the optimal number of principal components.

**Figure 6:**
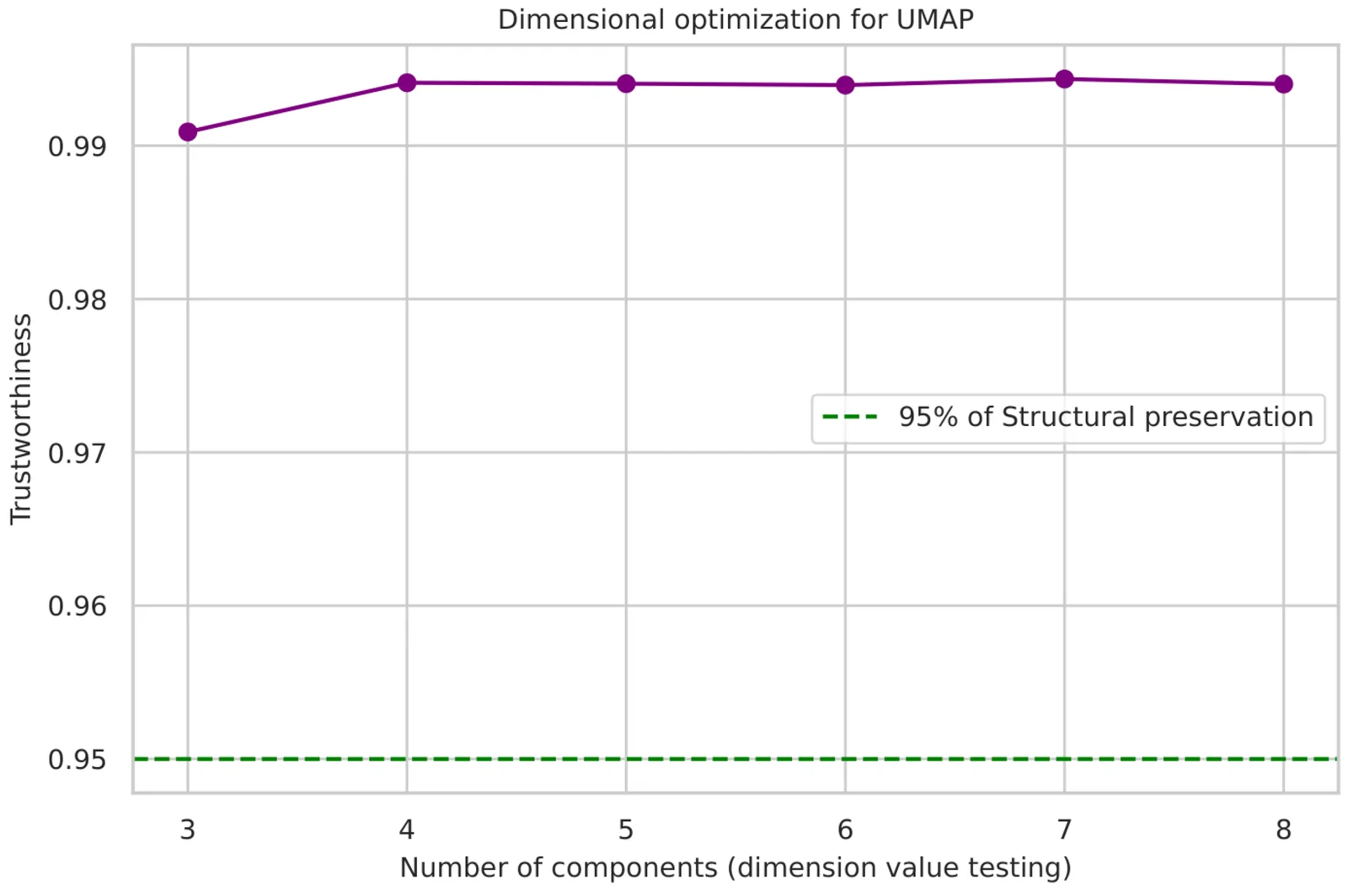
Selection of the optimal UMAP embedding dimensionality.

**Figure 7:**
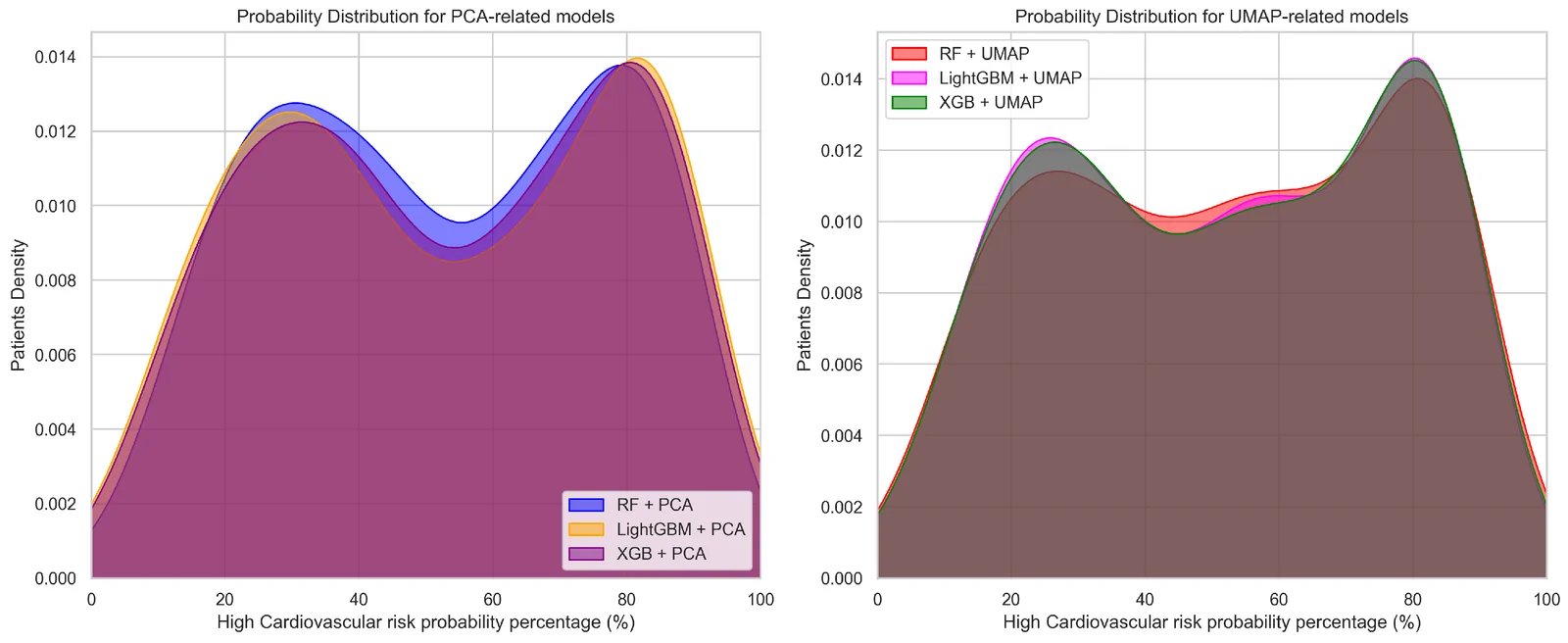
Comparative posterior probability distributions for PCA- and UMAP-based ensemble classifiers. The two panels use independent vertical (density) axes and should therefore be read as within-panel distribution shapes rather than compared by absolute height across panels.

**Figure 8:**
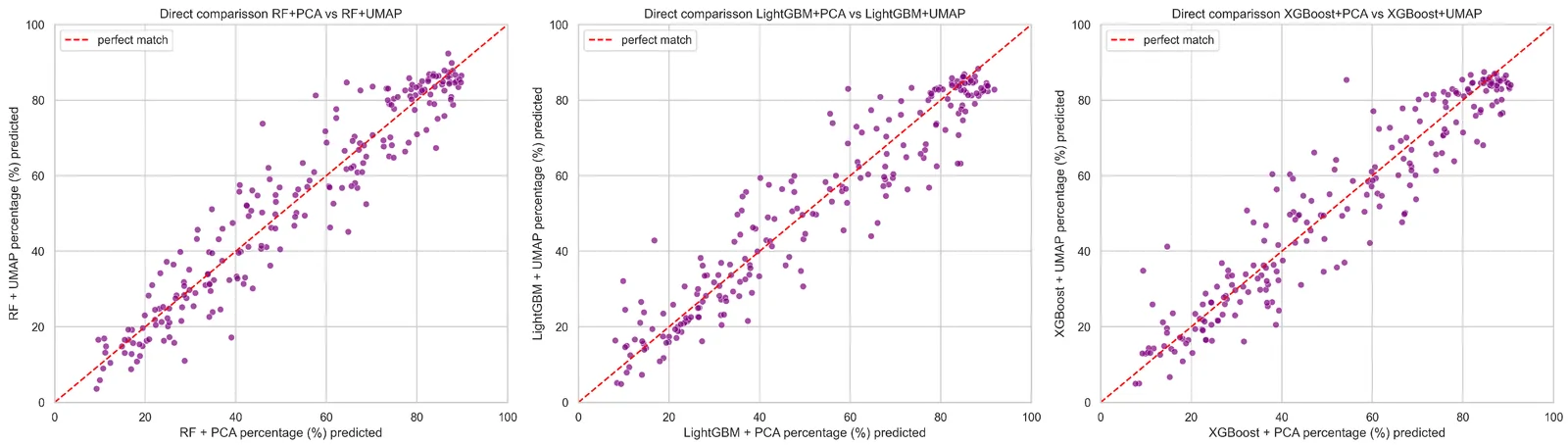
Pairwise concordance analysis between PCA- and UMAP-based probability assignments.

**Figure 9:**
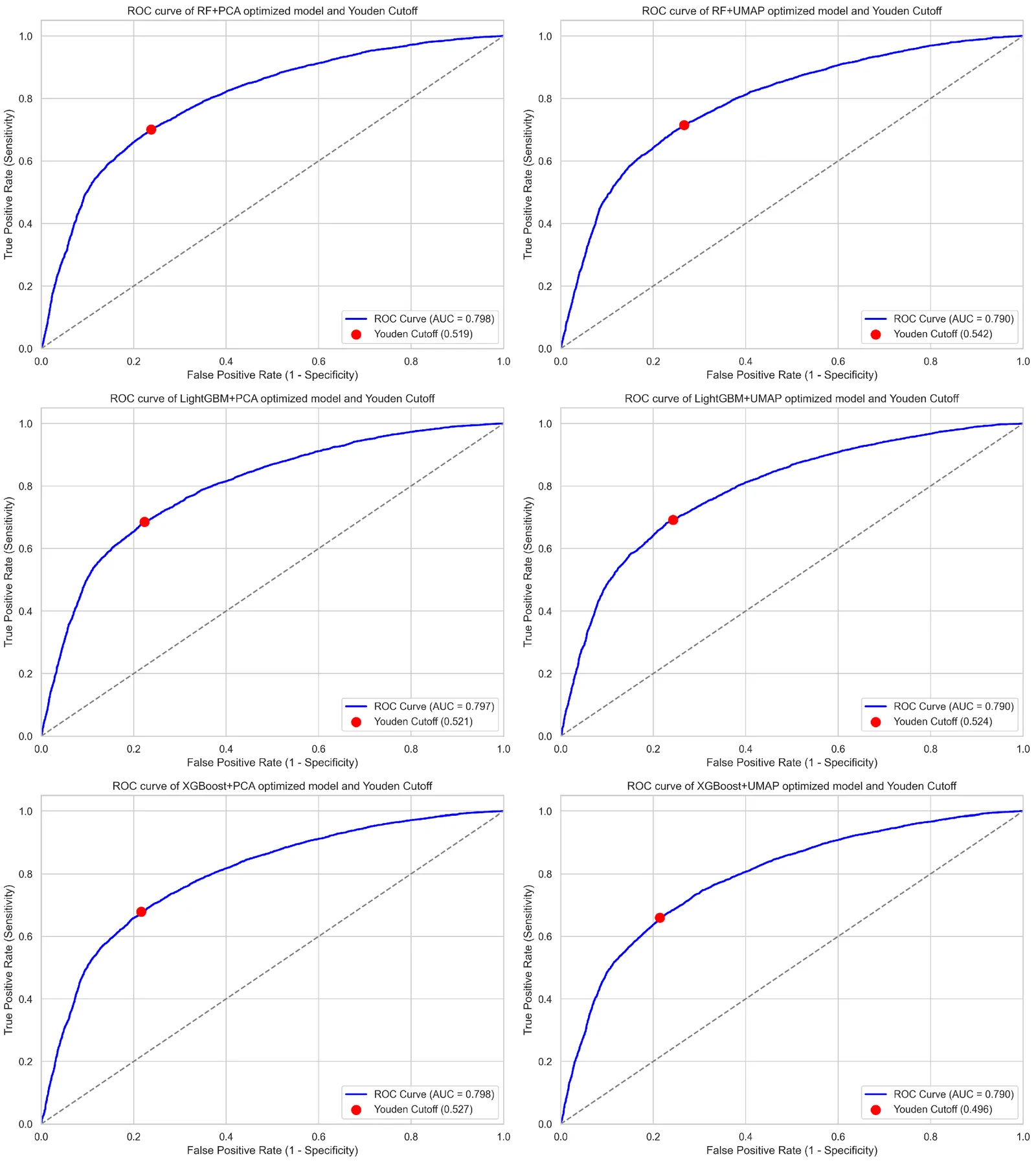
Receiver operating characteristic curves for the six integrated classification pipelines. The corresponding area-under-the-curve (ROC-AUC) values are reported per pipeline in Table 2 (training cohort ≈0.79) and, for the external cohort, in Table 3 (AUC_ext_ 0.82–0.85).

**Figure 10:**
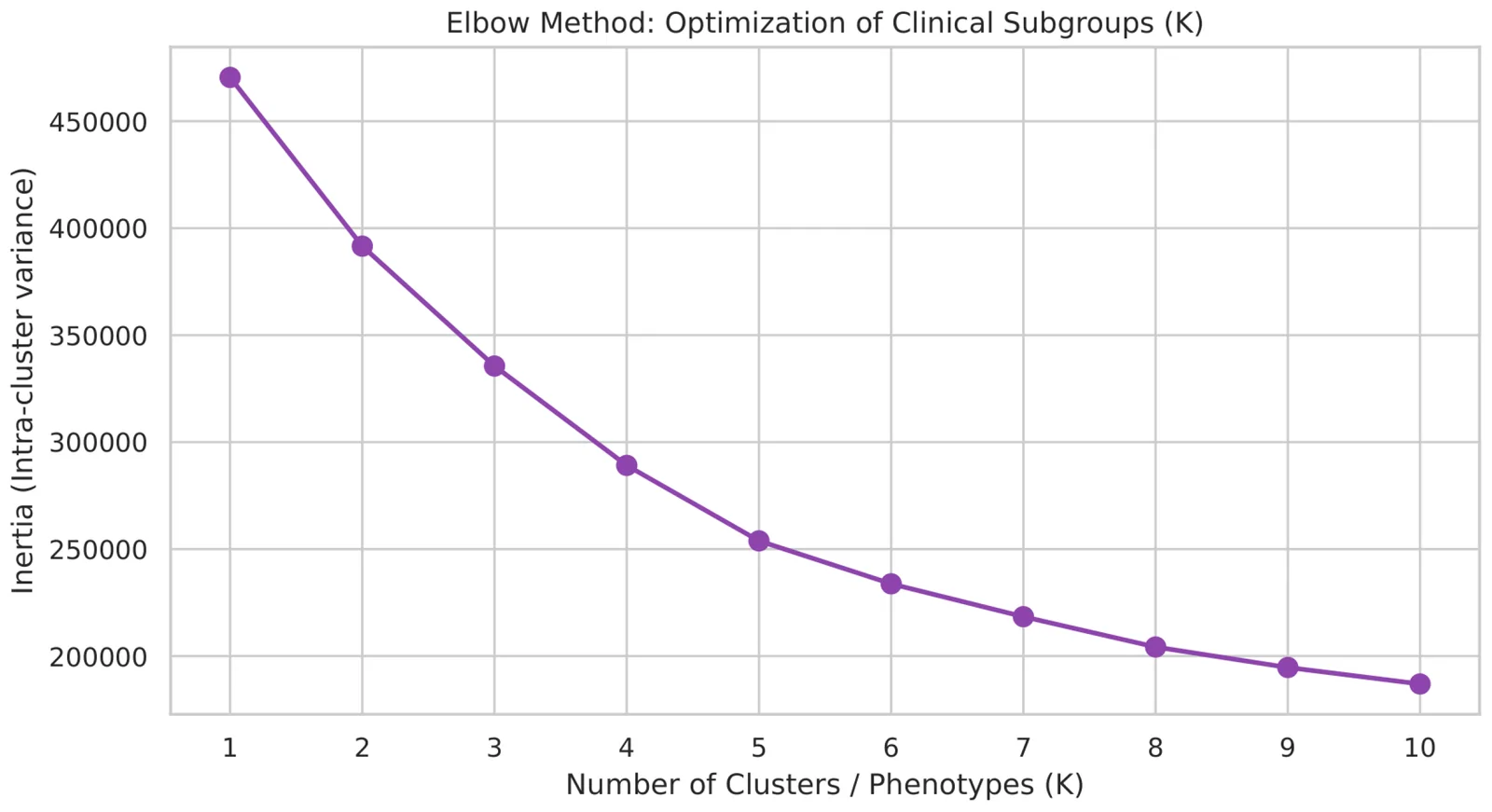
Elbow method for determining the optimal number of clusters.

**Table 1:** Comparative performance of nine classification architectures evaluated on the cardiovascular risk stratification task. All F1 columns report the *macro*- averaged F1-score (unweighted mean across the four risk classes): “F1 val.” and “F1 test” are computed on the held-out validation and test partitions, and “CV F1” is the mean macro F1 across cross-validation folds; ROC-AUC is the one-vs-rest macro-averaged area under the curve. Macro F1-score on the validation set was used as the primary selection criterion.

Model	F1 Val.	F1 Test	CV F1	ROC-AUC
Logistic Regression	0.7051	0.6860	0.8645	0.9877
Decision Tree	0.9724	0.9777	0.9910	0.9885
Random Forest	0.9837	0.9614	0.9947	0.9999
Gradient Boosting	0.9721	0.9674	0.9939	0.9988
**LightGBM**	**0.9870**	**0.9733**	**0.9954**	**0.9999**
CatBoost	0.9724	0.9772	0.9952	0.9999
SVM (RBF)	0.8147	0.8229	0.8834	0.9928
k-NN (k=7)	0.6902	0.7282	0.8805	0.9390
MLP (128-64-32)	0.9552	0.9561	0.9874	0.9998

**Table 2:** Comparative performance of the six integrated classification architectures.

Model	Accuracy	F1 (0)	F1 (1)	Threshold	ROC-AUC
Random Forest + PCA	0.7313	0.74	0.72	0.5186	0.7978
Random Forest + UMAP	0.7253	0.74	0.71	0.5421	0.7901
LightGBM + PCA	0.7289	0.74	0.72	0.5214	0.7970
LightGBM + UMAP	0.7222	0.74	0.71	0.5239	0.7914
XGBoost + PCA	0.7302	0.74	0.72	0.5027	0.7979
XGBoost + UMAP	0.7272	0.73	0.72	0.4958	0.7920

**Table 3:** Performance of the six integrated pipelines on the external validation cohort (n=219; 114 CVD-positive). Accuracy is reported with a 95% bootstrap confidence interval (3000 resamples). Sens, sensitivity; Spec, specificity; PPV/NPV, positive/negative predictive value; Bal. Acc, balanced accuracy; AUC_ext_, area under the ROC curve on the external cohort; Brier, Brier score. Thr. is the training-derived Youden cutoff, listed per pipeline and applied unchanged to the external cohort.

Model	Thr.	Hits	Acc. % [95% CI]	Sens	Spec	Bal. Acc	F1	AUC_ext_	Brier
LightGBM + PCA	0.5214	157	71.7 [65.8, 77.6]	71.1	72.4	71.7	0.713	0.835	0.169
LightGBM + UMAP	0.5239	160	73.1 [67.1, 79.0]	73.7	72.4	73.0	0.733	0.827	0.174
XGBoost + PCA	0.5027	158	72.1 [66.7, 78.1]	72.8	71.4	72.1	0.722	0.835	0.169
XGBoost + UMAP	0.4958	154	70.3 [64.4, 76.7]	75.4	64.8	70.1	0.712	0.821	0.175
Random Forest + PCA	0.5186	161	73.5 [67.6, 79.5]	72.8	74.3	73.5	0.732	0.846	0.165
Random Forest + UMAP	0.5421	163	74.4 [68.9, 79.9]	72.8	76.2	74.5	0.738	0.839	0.168

**Table 4:** Descriptive statistics of filtered training cohort by phenotype. Continuous variables are cluster means and categorical variables are the modal (most frequent) category. The CVD row reports the true prevalence of the outcome and the raw counts ($n^{+}/n$) per cluster. Gender is coded as in the source Kaggle dataset (1 = female, 2 = male); cholesterol and glucose are ordinal levels 1 (normal), 2 (above normal), 3 (well above normal).

Variable	Phenotype 0	Phenotype 1	Phenotype 2	Phenotype 3	Phenotype 4
Age	52.89	52.44	55.88	53.17	53.29
Height	1.71	1.68	1.63	1.61	1.64
Weight	77.43	78.18	79.10	71.21	73.39
Systolic BP	128.05	129.15	133.51	125.16	126.13
Gender	2.00	2.00	1.00	1.00	1.00
Cholesterol	1.00	1.00	3.00	1.00	1.00
Glucose	1.00	1.00	3.00	1.00	1.00
Smoking	0.00	1.00	0.00	0.00	0.00
Alcohol intake	0.00	1.00	0.00	0.00	0.00
Physically active	1.00	1.00	1.00	1.00	0.00
CVD prevalence	0.4889	0.4762	0.6505	0.4681	0.5187
CVD cases ($n^{+}/n$)	6444/13,181	1411/2963	3546/5451	11683/24,956	4787/9228
n	13181	2963	5451	24956	9228

**Table 5:** Descriptive statistics of validation cohort by phenotype. Continuous variables are cluster means and categorical variables are the modal category; the CVD row reports the true prevalence and raw counts ($n^{+}/n$) per cluster. These clusters come from an independent K-means fit on the 219 external patients and are therefore not matched to the training phenotypes of Table 4; they are labeled V-cluster 1–5 to prevent cross-table comparison. Gender is coded as in the source Kaggle dataset (1 = female, 2 = male); cholesterol and glucose are ordinal levels 1 (normal), 2 (above normal), 3 (well above normal).

Variable	V-Cluster 1	V-Cluster 2	V-Cluster 3	V-Cluster 4	V-Cluster 5
Age	52.50	51.90	54.02	53.68	54.53
Height	1.72	1.61	1.65	1.68	1.62
Weight	76.41	67.39	75.93	70.05	79.72
Systolic BP	131.30	125.14	128.93	133.18	141.97
Gender	2.00	1.00	1.00	2.00	1.00
Cholesterol	1.00	1.00	1.00	1.00	3.00
Glucose	1.00	1.00	1.00	1.00	1.00
Smoking	0.00	0.00	0.00	1.00	0.00
Alcohol intake	0.00	0.00	0.00	0.00	0.00
Physically active	1.00	1.00	0.00	1.00	1.00
CVD prevalence	0.4545	0.4286	0.4773	0.5000	0.9062
CVD cases ($n^{+}/n$)	20/44	33/77	21/44	11/22	29/32
n	44	77	44	22	32

**Table 6:** Permutation test of the association between cluster assignment and the CVD outcome (2000 label permutations of cardio). NMI, normalized mutual information; “Null NMI” gives the mean and 95th percentile of the permutation null distribution. The reported $p=5\times 10^{-4}$ is the minimum attainable value with 2000 permutations (1/2000): the observed statistic fell beyond every permuted value in both cohorts. Note that, although statistically significant, the effect sizes are negligible.

Cohort	NMI	Cramér’s *V*	Null NMI (Mean, 95th Pct. )	*p*
Training (n=55,779)	0.0054	0.106	$4\times 10^{-5}$, $1\times 10^{-4}$	$5\times 10^{-4}$
Validation (n=219)	0.0538	0.323	0.0088, 0.0207	$5\times 10^{-4}$

## Data Availability

The datasets analyzed during the current study are publicly available in open-access Kaggle repositories, as cited in references [8,9,10]. No new primary data were generated during this research. Pre-existing dataset repositories were utilized solely to train and evaluate the machine learning architectures for predicting high-risk patient stratification based on the Area Under the Receiver Operating Characteristic Curve (AUC) metric, integrating both supervised learning and dimensionality reduction approaches.The performance metrics presented in the tables represent the primary data generated by this study. The complete source code, including preprocessing pipelines, model training scripts, random seeds, and library versions required to reproduce all reported results, is openly available at https://github.com/alpoca99/Cardiovascular-risk-classification-with-supervised-models-and-clustering.git and archived at Zenodo (DOI: https://doi.org/10.5281/zenodo.21830373 Last update: September 29th, 2026).

## Abbreviations

The following abbreviations are used in this manuscript:

CVD	Cardiovascular Disease
AI	Artificial Intelligence
ML	Machine Learning
KNN	K-Nearest Neighbors
PCA	Principal Component Analysis
UMAP	Uniform Manifold Approximation and Projection
SVM	Support Vector Machine
RF	Random Forest
XGBoost	Extreme Gradient Boosting
LightGBM	Light Gradient Boosting Machine
GOSS	Gradient-based One-Side Sampling
EFB	Exclusive Feature Bundling
MEWS	Modified Early Warning Score
SMOTE	Synthetic Minority Oversampling Technique
ROC	Receiver Operating Characteristic
AUC	Area Under the Curve
CART	Classification and Regression Trees
ANN	Artificial Neural Network
HCA	Hierarchical Cluster Analysis
DBSCAN	Density-Based Spatial Clustering of Applications with Noise
OOB	Out-of-Bag
BP	Blood Pressure
ap_hi	Systolic blood pressure
ap_lo	Diastolic blood pressure
gluc	Glucose
alco	Alcohol Intake
active	Physical activity
cardio	Presence or absence of cardiovascular disease (1,0)

## Appendix A. KNN Preprocessing Pipeline Specifications

Once the optimal *k* is identified (Figure 3), the preprocessing pipeline is retrained with this hyperparameter on the primary Kaggle cohort and serialized to a portable binary format via Python’s pickle protocol. This allows the entire preprocessing architecture, including the fitted internal states of the normalization and imputation components, to be redeployed in later phases without retraining.

The imputation follows three steps. First, the Euclidean distance between the target observation and every candidate is computed only over the dimensions where both records are non-null:

(A1) $$d\left(x,x_{i}\right)=\sqrt{\sum_{j\in O_{i}}{\left(x_{j}-x_{ij}\right)}^{2}},$$

where $O_{i}$ denotes the set of feature dimensions on which both *x* and $x_{i}$ are observed (non-null), so that the distance is computed only over jointly observed coordinates. Second, the *k* instances with the smallest distances are selected. Third, missing continuous variables are imputed with the arithmetic mean of the neighbors, and categorical variables with their mode. Because the KNNImputer operates on a continuous domain, its estimates for discrete features are subsequently mapped to the nearest valid integer, ensuring the imputed data respect real-world clinical categories. A boolean missingness mask restricts imputation to the masked coordinates, leaving observed metrics unaltered.

Finally, the encoding strategy was tailored to the classifiers: for tree-based ensembles (Random Forest and XGBoost), Label Encoding maps each category to a unique integer, which is appropriate because decision-tree induction proceeds by recursive binary partitioning and does not presuppose distance-based relationships between feature values [19].

## Appendix B. Random Forest (RF) Algorithm

Random Forest (RF) is a supervised ensemble method that trains many decision trees on bootstrap samples of the data and aggregates their outputs, by majority vote for classification or averaging for regression, to produce a stable prediction [20]. Because individual trees are highly sensitive to fluctuations in the training set and prone to overfitting, this ensembling substantially reduces variance and improves generalization [21]. RF is well suited to non-linear modeling and variable-importance assessment, though it can be computationally costly and less suited to purely descriptive analysis [5].

RF handles missing data either natively, through an out-of-bag proximity matrix, or through an external KNN imputer as used in this study [15]. Categorical features are mapped according to their scale: one-hot encoding for nominal variables, ordinal encoding for ordered clinical ranges, and target binarization to collapse multi-stage severity scales into a binary outcome [22]. The framework also provides intrinsic feature selection via impurity-based importance, isolating the attributes that account for most of the predictive signal while a correlation filter removes redundant variables [23,24]. To preserve the epidemiological prevalence of cardiovascular pathology across partitions, stratified *K*-fold cross-validation can be applied so that each fold mirrors the target class distribution [25].

## Appendix C. Extreme Gradient Boosting (XGBoost) Algorithm

XGBoost is a scalable gradient-boosted tree ensemble well suited to clinical prediction because it models sparse, complex, and non-linear patterns efficiently, aided by a weighted quantile sketch for approximate split finding [16,26]. Since many physiological variables relate to outcomes through thresholds and categorical hierarchies rather than linear proportionality, its capacity to capture such non-linearities makes it a strong choice over traditional regression, particularly on large, complex datasets [27].

The model predicts through *K* additive tree functions:

(A2) $${\hat{y}}_{i}=\phi \left(x_{i}\right)=\sum_{k=1}^{K}\;f_{k}\left(x_{i}\right),\;\;\;\;\;f_{k}\in F$$

where $x_{i}\in R^{m}$ is the input vector and F the space of regression trees (CART), each $f_{k}$ a tree with its own leaf weights. Trees are added sequentially to reduce the errors of their predecessors via gradient boosting, minimizing at iteration *t* the regularized objective

(A3) $$L^{\left(t\right)}=\sum_{i=1}^{n}\;l\left(y_{i},\;{\hat{y}}_{i}^{\left(t-1\right)}+f_{t}\left(x_{i}\right)\right)+\Omega \left(f_{t}\right),$$

where *l* is a differentiable convex loss quantifying the gap between prediction and target, and Ω penalizes model complexity to prevent overfitting. Considering Equation (A2), ${\hat{y}}_{i}^{\left(t\right)}$ is the ensemble prediction for instance *i* at iteration *t*, adding $f_{t}$ to minimize $L^{\left(t\right)}$ [26].

## Appendix D. Light Gradient Boosting Machine (LightGBM) Algorithm

LightGBM is a gradient-boosting framework that builds decision trees sequentially, each minimizing the residual errors of its predecessors, with algorithmic innovations designed for large, high-dimensional data [12]. Two techniques underpin its efficiency: Gradient-based One-Side Sampling (GOSS), which keeps all high-gradient instances while subsampling low-gradient ones under a corrective weight that preserves the data distribution, and Exclusive Feature Bundling (EFB), which merges mutually exclusive sparse features into single composite variables to reduce memory and runtime [12,28]. These properties are well matched to the heterogeneous cardiovascular data, which combine dense continuous physiological metrics with sparse categorical features drawn from the combined clinical cohorts analyzed in this study.

Unlike the level-wise growth of conventional boosting, LightGBM grows leaf-wise, at each step splitting the leaf that most reduces the loss, independently of depth [12,28]:

(A4) $$j^{*}=\arg\max_{j\in L}\Delta L_{j},$$

where L is the set of active leaves and $\Delta L_{j}$ the objective gain from splitting leaf *j*. This yields lower training loss for a given number of leaves but requires regularization (chiefly num_leaves) to avoid overfitting on smaller datasets. In this study, LightGBM’s efficiency on large, heterogeneous cohorts complements XGBoost, whose exact greedy split-finding and conservative defaults make it more robust on smaller, well-scaled datasets [28,29]. Although LightGBM can handle categorical variables natively, label encoding was applied uniformly across all classifiers to maintain methodological consistency [12].

## Appendix E. Principal Component Analysis (PCA)

PCA is a linear dimensionality-reduction technique that transforms correlated clinical variables into a compact set of orthogonal, uncorrelated principal components, filtering noise and multicollinearity while retaining most of the original variance [5,11]. It identifies the directions of maximal variance, treating these as the primary underlying dynamics and low-variance axes as noise [30,31]. Its main drawbacks are limited clinical interpretability, since components are abstract linear combinations rather than physical metrics, and sensitivity to outliers and non-linear structure, which requires prior feature scaling [32].

Formally, the empirical covariance matrix of the mean-centered data *X* over *n* observations,

(A5) $$C=\frac{1}{n-1}\;XX^{T},$$

is subjected to an eigendecomposition seeking eigenvectors *w* and eigenvalues λ such that Cw=λw [33]. The eigenvectors define the new basis, and the data are projected via T=XW, where *W* is the eigenvector matrix and *T* the principal-component scores.

## Appendix F. Uniform Manifold Approximation and Projection (UMAP)

UMAP is a non-linear dimensionality-reduction algorithm grounded in Riemannian geometry and algebraic topology [34]. Unlike PCA, which seeks a global linear projection maximizing variance, UMAP assumes the data lie on a locally connected manifold whose topological structure is the quantity to preserve, and it builds an independent local metric around each point so that its *k* nearest neighbors appear uniformly distributed. These incompatible local spaces are merged into a single global fuzzy topological representation [34].

For each point, UMAP defines a directed edge weight to each neighbor $x_{ij}$,

(A6) $$w\left(x_{i},x_{ij}\right)=\exp\;\left(\frac{-\max(0,\;d\left(x_{i},x_{ij}\right)-\rho_{i})}{\sigma_{i}}\right),$$

where $\rho_{i}$ is the distance to the nearest neighbor (enforcing local connectivity) and $\sigma_{i}$ a per-point smoothing factor; these directed weights are symmetrized into an undirected graph via the probabilistic t-conorm. A low-dimensional embedding $Y\subset R^{d}$ is then optimized so that its induced fuzzy graph $\left\{w_{ij}\right\}$ matches the high-dimensional graph $\left\{v_{ij}\right\}$, where $v_{ij}$ is the symmetrized edge weight between points *i* and *j* in the high-dimensional input space (obtained from the directed weights above via the probabilistic t-conorm) and $w_{ij}$ is the corresponding edge weight computed from the low-dimensional embedding coordinates; the objective is minimized by minimizing the fuzzy-set cross-entropy

(A7) $$C_{\mathrm{UMAP}}=\sum_{i\neq j}\left[v_{ij}\log\;\left(\frac{v_{ij}}{w_{ij}}\right)+\left(1-v_{ij}\right)\log\;\left(\frac{1-v_{ij}}{1-w_{ij}}\right)\right],$$

solved through stochastic gradient descent with negative sampling [34]. By preserving both local and global structure, the fitted transform projects out-of-sample clinical vectors onto the same manifold, which supports the external-validation behavior discussed in the main text.
